# Predicting immunotherapy response in melanoma using a novel tumor immunological phenotype-related gene index

**DOI:** 10.3389/fimmu.2024.1343425

**Published:** 2024-03-20

**Authors:** Shaoluan Zheng, Anqi He, Chenxi Chen, Jianying Gu, Chuanyuan Wei, Zhiwei Chen, Jiaqi Liu

**Affiliations:** ^1^ Department of Plastic and Reconstructive Surgery, Zhongshan Hospital (Xiamen), Fudan University, Xiamen, China; ^2^ Department of Plastic and Reconstructive Surgery, Zhongshan Hospital, Fudan University, Shanghai, China; ^3^ Artificial Intelligence Center for Plastic Surgery and Cutaneous Soft Tissue Cancers, Zhongshan Hospital, Fudan University, Shanghai, China; ^4^ Big Data and Artificial Intelligence Center, Zhongshan Hospital, Fudan University, Shanghai, China

**Keywords:** melanoma, bioinformatics, prognosis, immunotherapy response, tumor microenvironment, molecular docking

## Abstract

**Introduction:**

Melanoma is a highly aggressive and recurrent form of skin cancer, posing challenges in prognosis and therapy prediction.

**Methods:**

In this study, we developed a novel TIPRGPI consisting of 20 genes using Univariate Cox regression and the LASSO algorithm. The high and low-risk groups based on TIPRGPI exhibited distinct mutation profiles, hallmark pathways, and immune cell infiltration in the tumor microenvironment.

**Results:**

Notably, significant differences in tumor immunogenicity and TIDE were observed between the risk groups, suggesting a better response to immune checkpoint blockade therapy in the low-TIPRGPI group. Additionally, molecular docking predicted 10 potential drugs that bind to the core target, PTPRC, of the TIPRGPI signature.

**Discussion:**

Our findings highlight the reliability of TIPRGPI as a prognostic signature and its potential application in risk classification, immunotherapy response prediction, and drug candidate identification for melanoma treatment. The "TIP genes" guided strategy presented in this study may have implications beyond melanoma and could be applied to other cancer types.

## Introduction

Melanoma is a type of skin cancer that arise from melanocytes, the pigment-producing cells found in the epidermis, hair follicles, and iris, among other tissues (1). Melanoma only accounts for only 1% of skin cancers, but it is the most aggressive and dangerous one and accounts for 90% of all skin cancer deaths, melanoma patients with distant metastases show a 5-year survival rate of 23% (2, 3).

Melanoma is one of the most immunological malignancies (4, 5)., immunotherapy is one of the most effective therapeutic strategies in melanoma due to the high immunogenicity of this tumor (6). Immune checkpoint blockade (ICB) immunotherapy, which reverses the inactivation of immune cells to eliminate tumor cells, has emerged as a promising therapy for melanoma (7). In recent years, the immunologic origin of melanoma has led to the discovery of antibodies directed against specific targets such as anti-programmed cell death 1 (PD-1) and anti-cytotoxic T-lymphocyte-associated protein 4 (CTLA-4) (8). Since then, additional agents targeting novel immune checkpoints, such as T-cell immunoglobulin and mucin domain containing 3 (TIM-3), lymphocyte activation gene-3 (LAG-3), and ITIM domain (TIGIT), are being investigated to expand the scope of immunotherapy for melanoma (9–11).

Overall, these blockades have dramatically increased and prolonged overall survival (OS) in advanced melanoma. However, even in the most optimal scenarios with a combination ICB, approximately half of the patients do not achieve a long-term benefit (12). While elevated tumor PD-L1 expression and TMB have been shown to correlate with clinical response to ICB in melanoma, these biomarkers may not accurately predict outcome in all cases (13). This highlights the need for better predictive biomarkers of response and new rational targets for more effective combination therapies to overcome immune resistance. How to choose available and suitable targets for personalized therapy is still a challenging question.

Tumor microenvironment (TME) encompasses a wide range of stromal, vascular, and immune cell types that are impacted by therapy. The TME is considered one of the primary indicators of treatment response in cancer due to its high level of heterogeneity (14). Tumor immune phenotype (TIP) is an emerging concept to evaluate immunologic heterogeneity depending on the relative infiltration of immune cells, and tumors are generally classified into two TIPs: “hot” (inflamed) and “cold” (non-inflamed), respectively (15). Oncogenic genetic and epigenetic pathways simultaneously define TIPs, influence tumor progression, and alter spontaneous and therapy-induced tumor-specific T cell immunity. Manipulation of these tumor intrinsic pathways can promote T cell infiltration into tumors, alter the tumor immune phenotype and ultimately lead to tumor regression. Thus, these “TIP genes” offer significant potential for clinical application, particularly in the areas of postoperative risk stratification and identification of immunotherapeutic predictors in cancers. The immunotherapy prediction model established based on TIP has been validated in hepatocellular carcinoma (16), but has not been investigated in melanoma.

**Figure 1:**
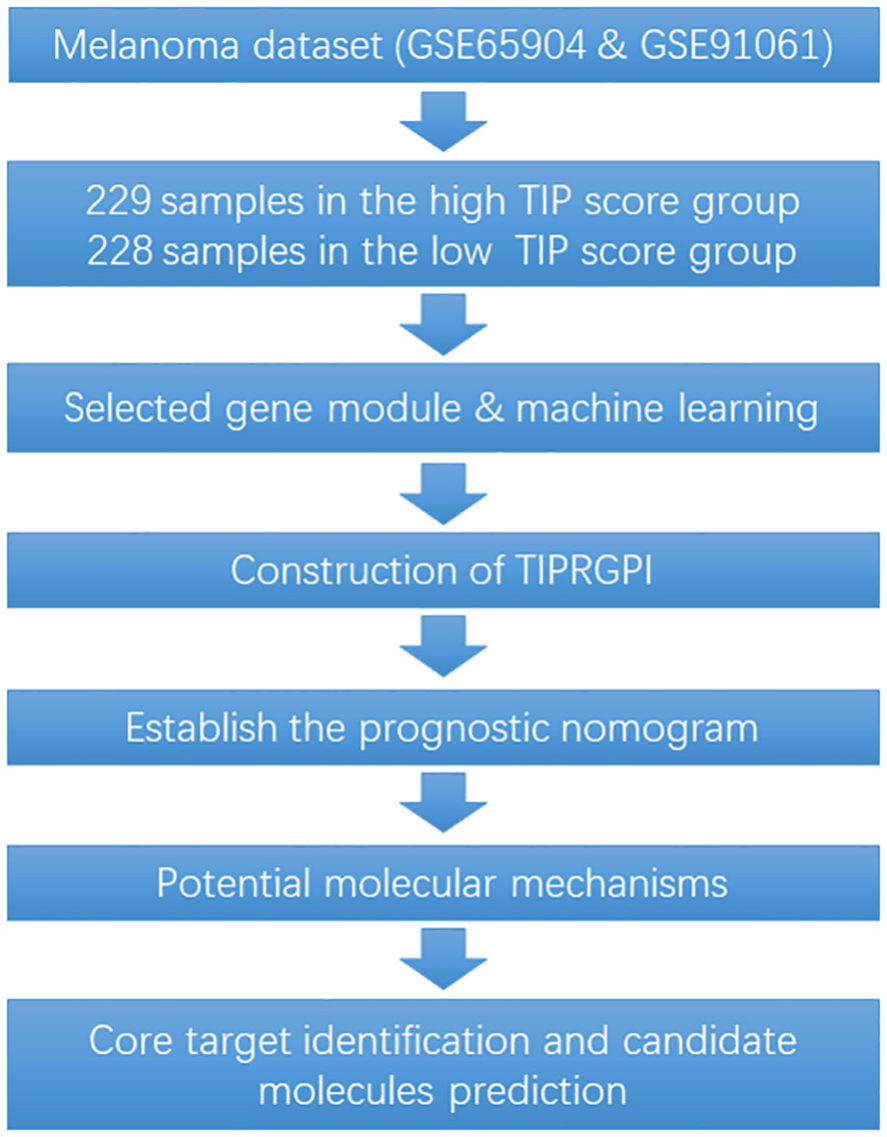
The workflow for this study.

To fill the gap between TIP-based prediction model and immunotherapy response in melanoma, in this study, a “TIP genes” guided strategy was utilized with various statistical algorithms to create a TIP-related gene prognostic index (TIPRGPI), which is a novel melanoma signature. It is predicted that individuals in the low-risk TIPRGPI group will respond more favorably to immunotherapies, as compared to those in the high-risk TIPRGPI group. Furthermore, based on our molecular docking analysis, we have identified 10 potential drugs that successfully bind to the core target of TIPRGPI. The workflow for this study is shown in **Figure 1**.

## Materials and methods

### Data source

Download RNA sequencing, phenotype, and survival data for skin melanoma (SKCM) from the TCGA database at https://xenabrowser.net/datapages/. Obtain skin melanoma gene expression RNA sequencing and survival information data from GSE65904 at https://www.ncbi.nlm.nih.gov/geo/query/acc.cgi?acc=GSE65904, and melanoma gene expression RNA sequencing, survival, and immunotherapy information data from GSE91061 at https://www.ncbi.nlm.nih.gov/geo/query/acc.cgi?acc=GSE91061. Hot and cold tumor genes were acquired from previous literature (16). The Gencode annotation files were acquired from the EBI website ftp://ftp.ebi.ac.uk/pub/databases/gencode/Gencode_human/release_38/gencode.v38.chr_patch_hapl_scaff.annotation.gtf.gz. The gene set for the hallmark pathway, h.all.v7.5.symbols.gmt, was acquired from the MsigDB database found at http://www.gsea-msigdb.org/gsea/msigdb/. The TCGA and GEO datasets are shown in **Tables 1**–**3**.

**Table 1:** Clinical information on TCGA-SKCM.

Variable	Classic	No.
Sample type	Metastatic	361
	Primary Tumor	99
Age	≥60	217
	<60	246
Stage	Stage 0	6
	Stage I	78
	Stage II	136
	Stage III	174
	Stage IV	23
Pathologic T	T0	23
	T1	32
	T2	77
	T3	91
	T4	150
	Tis	7
	Tx	45
Pathologic N	N0	227
	N1	75
	N2	50
	N3	57
	Nx	34
Pathologic M	M0	411
	M1	24
Mutational Subtype	BRAF	147
	NF1	27
	RAS	91
	Triple Wild Type	48
Gender	Male	289
	Female	174
Breslow depth	<1.5	99
	1.5-3	68
	3-4.5	64
	>4.5	124
Melanoma clark level	I	5
	II	18
	III	77
	IV	164
	V	54
BMI* exposures	<18.5	3
	18.5-23.9	60
	>23.9	178

*BMI, body mass index.

**Table 2:** Clinical information on dataset GSE65904.

Variable	Classic	No.
Gender	Male	124
	Female	89
	Default	1
Age	≥60	130
	<60	80
	Default	4
Tissue	Cutaneous	22
	Lymph node	130
	Subcutaneous	33
	Visceral	10
	Other	1
	Default	18

**Table 3:** Clinical information on dataset GSE91061.

Variable	Classic	No.
Mutational Subtype	BRAF	28
	NF1	5
	RAS	18
	Triple Wild Type	41
	Default	17
M Stage	M0	2
	M1a	21
	M1b	17
	M1c	44
	Default	25
Treatment Response	CR	6
	NE	3
	PD	44
	PR	14
	SD	34
	Default	8

### Correlations of TIP score with prognosis and TME of melanoma

1. TIP score was calculated as previously reported (17). The study analyzed the expression levels of genes associated with hot tumors (CXCR3, CXCR4, CXCL9, CXCL10, CXCL11, CCL5, CD3E, CD4, CD8A, CD8B, CD274, and PDCD1) and cold tumors (CXCL1, CXCL2, and CCL20). Each gene’s z-value was calculated in its respective dimension, and all z-values were summed up in the sample dimension, resulting in the TIP score for each sample. Subsequently, the samples were grouped into high and low TIP score categories, with the median acting as the dividing line.
2. Analysis of TIP score and immune-related indicators. Calculated Immune Score, Stromal Score, ESTIMATE Score, and Tumor Purity using the estimate package (version 1.0.13). Also calculated CD4/CD8+ T cell score using the xCell package (version 1.1.0). Furthermore, the correlation between TIP score, Immune Score, Stromal Score, ESTIMATE Score, Tumor Purity, CD4/CD8+ T cell score, and PD-1/CTLA-4 expression was computed and visualized using the ggpubr package (version 0.4.0).
3. Analysis of TIP score correlation with other immunotherapy signatures. TIDE values were determined via the TIDE algorithm (http://tide.dfci.harvard.edu/). TIP score was computed and mapped against TMD and TIDE correlations utilizing the ggpubr package.

### TIPRGPI establishment

1. Screening of Key Modules Associated with TIP score Traits Based on Expression Profiling and Weighted Gene Co-Expression Network Analysis (WGCNA). The WGCNA package, version 1.69, screened for key modules that were significantly associated with TIP score, using TCGA-SKCM expression profiles (RsquaredCut=0.8, power=6, MM>0.5, GS>0.05). The resulting set of concatenated genes was then taken as the key module genes for subsequent analysis.
2. Gene Ontology (GO) enrichment and Kyoto Encyclopedia of Genes and Genomes (KEGG) pathway analysis were performed on the most significant module using the “clusterProfiler” R package. Plotting using the ggplot2 package (v3.3.5) and the enrichplot software (v1.14.2).
3. Survival package was utilized in performing Cox regression analysis on critical modular genes to identify genes related to prognosis. Additionally, glmnet package (version 4.1-1) was utilized in the LASSO regression analysis of prognosis-associated genes to eliminate redundant genes and to model them. Then, utilizing the survival package, K-M survival curves were generated for the modeled genes.

### TIPRGPI and outcome analysis

1. The clinical outcome in this study was overall survival (OS). The OS-predictive effectiveness of TIPRGPI was assessed in both the training set and an independent external dataset. Based on the median TIPRGPI in the training and validation sets, the samples were divided into two categories: high TIPRGPI and how TIPRGPI. Scatter plots depicting the variation in survival/death based on TIPRGPI score were generated utilizing the ggplot2 package, while heat maps illustrating the modeled gene expression differences between high and low TIPRGPI groups were generated utilizing the pheatmap package (version 1.0). 12) We generated survival curves for the high and low TIPRGPI groups using the survival package. Additionally, we plotted 1-3 years modeled ROC curves for these groups using the time-ROC (version 0.4) package.
2. To examine the predictive potential of TIPRGPI characteristics among various clinicopathological subgroups, survival variation diagrams comparing high and low TIPRGPI groups were generated for different age groups (<60 years, ≥60 years), genders, and stages (stage 0/I/II, stage III/IV) using the survival package.
3. Based on TCGA data, immune cell infiltration was determined using three algorithms, namely CIBERSORT, XCELL, and SSGSEA. The CIBERSORT (https://cibersortx.stanford.edu/), SSGSEA, and XCELL algorithms were utilized to assess the differences in cellular infiltration within the immune microenvironment and graphed using the ggplot2 package to compare the variances in cellular infiltration among various pyroptosis subtypes.
4. Heatmap displaying the variation in immune checkpoint expression among distinct pyroptosis subtypes, using the “pheatmap” software package.

### TIPRGPI-integrated predictive nomogram

1. The differences in the distribution of clinical variables such as age (<60 years, ≥60 years), gender, stage, TNM staging, TCGA molecular typing, primary/metastasis, and Clark level between high and low TIPRGPI groups were compared. Differences in the distribution of Breslow depth and BMI between the high and low TIPRGPI groups were also compared. The “complexHeatmap” package (version 2.6.2) was utilized for plotting purposes.
2. Various clinical factors were examined to evaluate disparities in TIPRGPI scores. The distinctions in TIPRGPI scores among subcategories of age, gender, stage, TNM staging, TCGA molecular typing, primary/metastasis, Clark level, Breslow depth, and BMI were compared and illustrated utilizing the ggpubr software package.
3. The single-factor cox regression utilized the survival package with variables such as age, gender, NM staging, and high/low TIPRGPI groupings. The relationship between variables such as age, gender, NM staging, and high/low TIPRGPI groupings were evaluated to reveal the independence of TIPRGPI as a prognostic factor for cutaneous melanoma. The analysis was visualized using forestplot (version 1.10.1). The results are presented in the forestplot.
4. Integrating clinical factors, the TIPRGPI is utilized to construct nomograms with column-line graphs for clinical analysis and predictive significance. The regplot package (version 1.1) is employed to plot age grouping and TIPRGPI grouping column line plots, while the rms package (version 6.1-0) is utilized to plot calibration curves. The rmda package (version 1.6) is used to plot decision curves, categorizing samples into high- and low-risk groups based on composite model scores. Finally, the survival package is deployed to compare and plot km curves between groups.

### Potential molecular mechanisms for TIPRGPI

1. Mutated gene analysis is performed to display co-mutation patterns in both high and low TIPRGPI groups. The maftools package (version 2.6.0) is utilized to generate SNV waterfall maps for both groups. The Hmisc package (version 4.4-2) calculates the top differential gene autocorrelation, and a heatmap is produced using the pheatmap package. Lastly, the maftools package is used to create differential gene lollipop plots.
2. CNV Differences Between High and Low TIPRGPI Groups, The CNV profiles of the samples in the high and low TIPRGPI groups were calculated on each chromosome using GISTIC from https://cloud.genepattern.org and plotted using the ggpubr package. Comparison of CNV differential genes between the groups was obtained and plotted using the ggplot2 package. Differences in the expression of CNV differential genes were compared and plotted using the ggpubr package, across CNV status, including amplification, normal, and deletion. The results were visualized in a violin plot.
3. Enrichment scores of hallmark pathways (h.all.v7.5.symbols) were computed for samples belonging to the high and low TIPRGPI groups employing the GSVA (version 1.38.0) and GSEABase (version 1.52.1) software packages. Differential genes were compared between the high and low TIPRGPI groups using the limma package. These genes were included in the GSEA analysis to obtain differentially enriched hallmark pathways, and the resulting pathways were compared with the GSVA results to obtain concordant result pathways. Finally, GSEA enrichment plots were plotted using the enrichplot package. The samples were categorized into high and low score groups based on the GSVA score of each consistent result pathway. Subsequently, survival curves were plotted for each pathway by using the survival package.

### Exploration of immune infiltration

1. Differences in tumor microenvironment cellular infiltration between high and low TIPRGPI groups. The infiltration scores of 24 cell types in the tumor microenvironment for samples from the high and low TIPRGPI groups were calculated using the ssGSEA algorithm and compared and plotted using the ggpubr package and the ggplot2 package.
2. The Hmisc package was used to calculate the differential correlation between TME cells and TIPRGPI.
3. TIPRGPI screening of cells most associated with TME. TME cells significantly associated with prognosis were screened. The ggvenn package (version 0.1.9) was used to plot Wayne plots of significant cells in the analysis of variance, correlation, and survival, and the cells that were significant in all 3 were taken as the cells most associated with TME by TIPRGPI. The number of cells in the 3 analyses was plotted using the ggplot2 package for bar graph display.
4. Correlations between the 20 model genes and the 21 most relevant TME cells were calculated using the Hmisc package, and heat maps were generated using the pheatmap package.
5. Differences in the expression of several immune gene sets were analyzed between the high and low TIPRGPI groups. Enrichment scores for samples in the high and low TIPRGPI groups on the immune-related 7 gene set were calculated using GSVA, and differences between groups were compared. Heatmaps of gene expression in the 7 gene sets were generated using pheatmap.

### Estimating TIPRGPI to predict immunotherapeutic response

1. According to previous publications, the correlations between TIPRGPI and potential immunotherapeutic markers including IFN-gamma pathway markers, m6A regulators, Immunophenoscore (IPS) scores and Tumor Immune Dysfunction and Exclusion (TIDE) values were explored. Differential box plots of expression differences of immune checkpoints, IFN-gamma pathway marker genes, and m6A regulators between high and low TIPRGPI groups were compared and plotted using the ggplot2 and ggpubr packages. TCGA-SKCM sample data were downloaded from https://tcia.at/patients using the ggdist (version 3.2.0), gghalves (version 0.1.3), ggsci (version 2.9), ggplot2, and ggplubr packages to plot various IPS differences between high and low TIPRGPI groups in the IPS subgroups in a cloud rain plot. TIDE values were calculated for the TCGA-SKCM samples using the TIDE algorithm (http://tide.dfci.harvard.edu/), and TIDE differences between the high and low TIPRGPI groups were compared and plotted using the ggplot2 and ggpubr packages.
2. TIPRGPI differences between groups were compared and plotted using the ggplot2 and ggpubr packages. ROC curves were plotted with the time-ROC package.

### TIPRGPI-related core target identification and candidate molecule prediction

To identify the core target of the TIPRGPI signature, all genes were uploaded to the online database of the Search Tool for the Retrieval of Interacting Genes (STRING) (version 11.0; http://string-db.org/) for protein-protein interaction (PPI) network construction with default settings (interaction score ≥0.4). Cytoscape (version 3.2.1; http://www.cytoscape.org) was used for visualization. Next, we calculated the topological parameters using the Network Analyzer plugin and obtained the degrees of all nodes in the network. The core target was identified as the node with the highest degree.

### Molecular docking

The structural information of the corresponding compounds was downloaded from the DrugBank database and screened according to Lipinski’s rule (hydrogen bond acceptor≤ 10, hydrogen bond donor ≤ 5, rotatable bond ≤ 10, logarithmic value of lipid-water partition coefficient ≤ 5, molecular weights of 180-480, and polar surface area ≤ 140), and finally 5462 small molecule compounds were obtained. Retrieve the spatial structure information of the key gene-encoded proteins in the PDB database, find the corresponding data from protein tyrosine phosphatase receptor type C (PTPRC), and download the corresponding PDB files.5FN7. Determine the approximate docking box range and set the other relevant parameters of autodock-vina, and dock with small molecule compounds using autodock-vina, and perform interaction force analysis using Pymol and Ligplus for interaction force analysis.

### Statistical analysis

All analyses and plotting in this study were conducted using R version 4.2.1. In detail, the R script can be downloaded from the GitHub (https://github.com/Myth1313/Melanoma-immunotherpy-prediction-model-based-on-TIP-.git). A significance level of P<0.05 was employed, with two-tailed Wilcoxon rank-sum tests for between-group differences comparisons, chi-square tests for frequency differences comparisons among multiple groups, and log2 fold change≥1.5 as the criteria for differential gene selection in Gene Set Enrichment Analysis (GSEA). COX survival analysis was performed using the R packages survival and survminer, with survival curves plotted using the Kaplan-Meier (K-M) method. LASSO analysis was employed to identify the most advantageous gene combinations for constructing TIPRGPI to present K-M curves. The receiver operating characteristic (ROC) curve was used and a larger area under the ROC curve (AUC) indicated a better predictive performance. An AUC of 0.9–1.0 is considered excellent, 0.8–0.9 very good, 0.7–0.8 good, 0.6–0.7 sufficient, 0.5–0.6 bad, and less than 0.5 considered not useful. (ns: p>0.05; *: p<=0.05; **: p<=0.01; ***: p<=0.001; ****: p<=0.0001). 

## Results

### TIP score was associated with the prognosis and the immune state of melanoma

**Figure 2:**
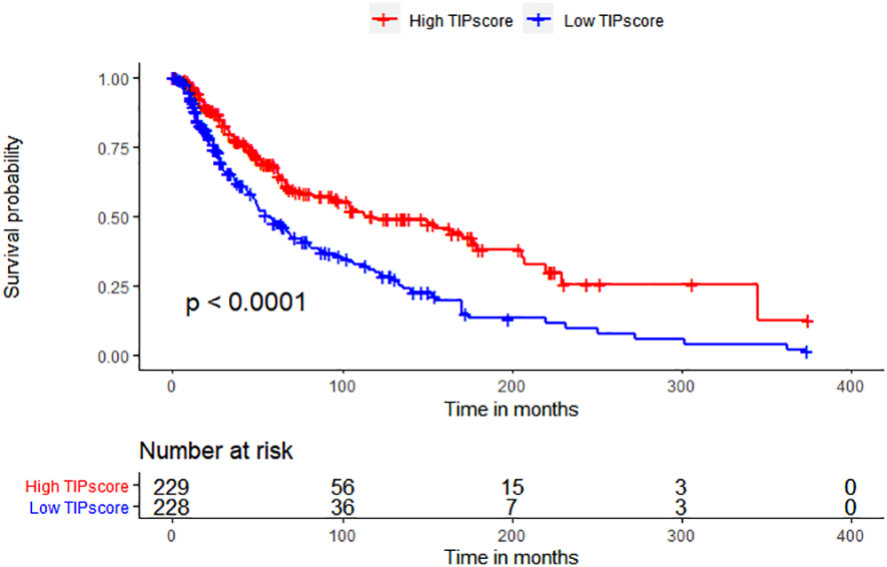
TIP score correlates with the prognosis and the immune state of melanoma. Kaplan–Meier survival plots of TIP score for OS.

To determine whether TIP score is effective in melanoma, we performed a series of survival analyses using Kaplan-Meier (K-M) survival curves and log-rank tests to examine the discrepancy between low and high TIP score groups. High and low TIP score groups were formed based on the median TIP score (median = -1.304159) with 229 samples in the high TIP score group and 228 samples in the low TIP score group (TIP score and grouping detailed in **Supplementary Table 1**). As shown in **Figure 2**, melanoma patients with higher TIP scores had a better prognosis. We then analyzed the correlations among TIP score, immune score, stromal score, estimate score, and tumor purity. The TIP score correlated positively with immune, stromal, and estimation scores, but negatively with tumor purity. Additionally, as effective T cells - including activated CD4 and CD8 T cells - are crucial in the tumor microenvironment, we computed their positive correlations with the TIP score. Furthermore, since PD-1 or CTLA-4 serve as essential immune checkpoints, we confirmed their positive correlation with the TIP score. The TIP score had strong linear correlations with Immunity score, ESTIMATE score, tumor purity, CD8+ T cell, and PD-1 checkpoints. TIP score correlations with TMB and TIDE were all statistically significant but weak (**Supplementary Figures 1**, **2**).

### Construction of TIPRGPI

**Figure 3:**
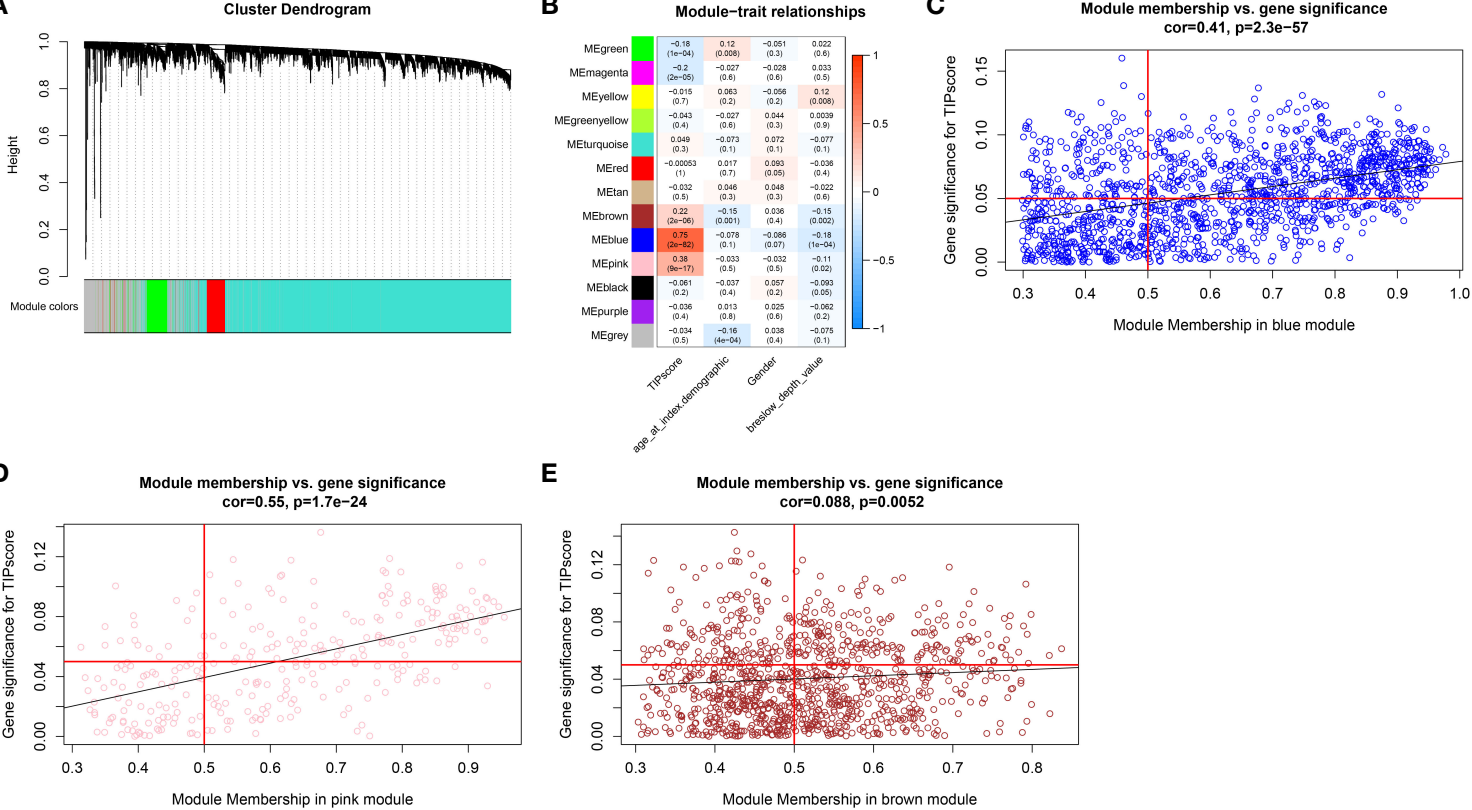
WGCNA analysis. **(A)** Cluster dendrogram of MAD top 5000 genes. **(B)** Table cells show Pearson correlation coefficients and corresponding P-values between module eigengenes (ME) and the variables in 13 modules. **(C)** Scatter plot depicting the correlation between gene significance (GS) for TIP score and module membership (MM) in the blue module. **(D)** Scatter plot depicting the correlation between GS for TIP score and MM in the pink module. **(E)** Scatter plot depicting the correlation between GS for TIP score and MM in the brown module.

**Figure 4:**
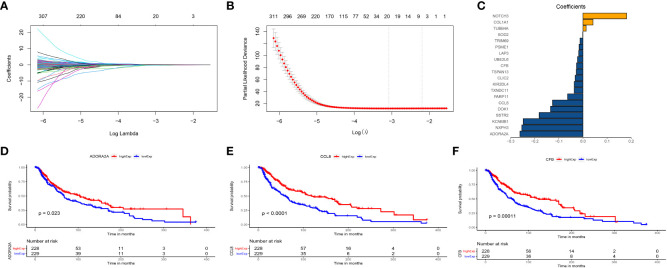
The construction of TIPRGPI for melanoma. **(A)** The penalty function for the LASSO algorithm was chosen as lambda.min = 0.04591217. **(B)** LASSO regression using the 128 genes. **(C)** Twenty robust genes were and their coefficients in the model. **(D)** ADORA2A positively correlated with OS. **(E)** CCL8 positively correlated with OS. **(F)** CFB positively correlated with OS.

WGCNA was used for the identification of the gene module associated with TIP scores. The resulting co-expression network revealed that genes were clustered into 13 modules, which we used to analyze the data (**Figure 3A**). We analyzed the correlation and significance of each module eigengenes (ME) with TIP score, age, gender, and Breslow depth using Pearson correlation coefficient (**Figure 3B**). We chose the blue, pink, and brown modules that exhibit the strongest and most significant correlation with TIP score. The scatter plots for the GS-MM of the three modules are shown in (**Figures 3C–E**). The genes in the upper right red grids of the three plots were selected to create a concatenation set as the key module genes related to TIP score (934), which can be found in **Supplementary Table 2**. The genes in blue, pink, and brown modules are primarily involved in biological process (BP) of T cell activation, cellular component (CC) of mononuclear cell proliferation, and molecular function (MF) of immune receptor activity. KEGG analysis indicates a predominant involvement in the cytokine-cytokine receptor interaction pathway (**Supplementary Figure 3**). The genes from the blue, pink, and brown modules were then inserted in a UniCox regression analysis and identified 641 significant genes (listed in **Supplementary Table 3**) with a P value lower than 0.05. We then performed LASSO regression using the 128 genes and obtained 20 robust genes. The penalty function for the LASSO algorithm was chosen as lambda.min=0.04591217 to eliminate extraneous genes. **Figures 4A, B** shown the process of removing redundant genes by LASSO to get the optimal combination of genes, and **Figure 4C** shown the 20 genes and their coefficients in the model, which is modeled as.

TIPRGPI = -0.0176318658536308×LAP3 + 0.181442498785852×NOTCH3-0.0160031827383823×PSME1 + 0.013328065983228×TUBB4A-0.0275390143073945×TSPAN13-0.126145101349642×CCL8 + 0.0414697345452162×COL1A1-0.063751548357349×PARP11-0.0010811360894315×SOD2-0.134743050717298×DOK1-0.261361794333436×ADORA2A-0.247626113868466×KCNMB1-0.0366480831303996×TXNDC11-0.0325307756953961×CLIC2-0.0227464875810033×UBE2L6-0.181697447499612×SSTR2-0.253663685898908×NXPH3-0.012429025784462×TRIM69-0.0365676747211779×KIR2DL4-0.026482536599118×CFB

**Figures 4D–F** shows the positive correlations of ADORA2A, CCL8 and CFB with OS, respectively.

### Evaluation and validation of the TIPRGPI signature

**Figure 5:**
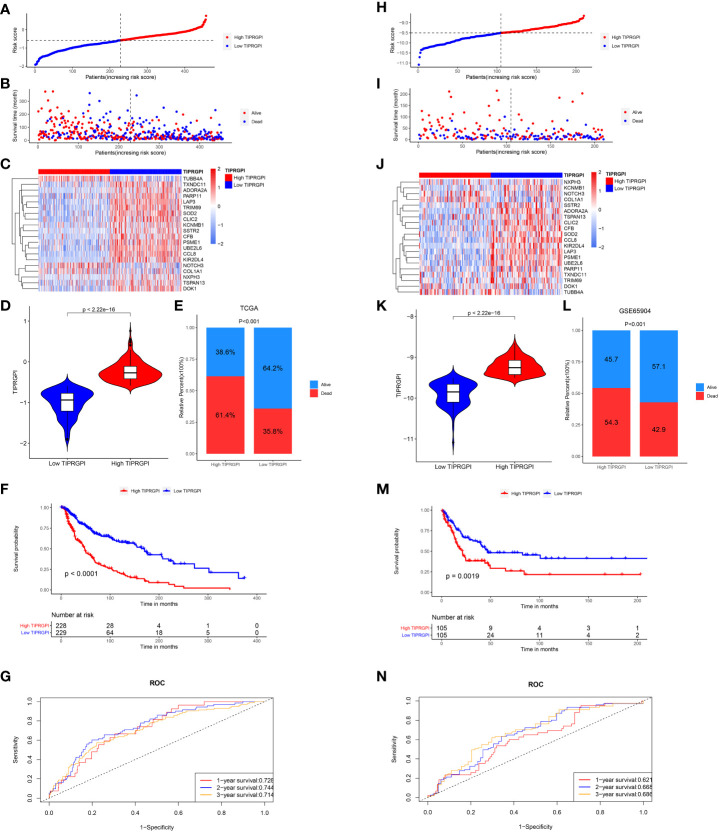
Validation of the TIPRGPI predicting model in melanoma. **(A–F)** Risk score distribution, survival status, the expression of 20 TIPRGPI genes, mortality rate Kaplan-Meier survival curves for patients in low- and high-TIPRGPI groups from training dataset TCGA-SKCM. **(G)** Time-dependent receiver operating characteristic (ROC) curves of training dataset. **(H–M)** Risk score distribution, survival status, the expression of 20 TIPRGPI genes, mortality rate Kaplan-Meier survival curves for patients in low- and high-TIPRGPI groups from validation dataset GSE65904. **(N)** Time-dependent receiver operating characteristic (ROC) curves of validation dataset.

The TIPRGPI score for each patient was calculated. Based on the median value from the training set, melanoma patients from TCGA-SKCM (training dataset, **Figure 5A**) and GSE65904 (validation dataset, **Figure 5H**) were classified into low- and high-risk groups. The survival time decreases as the score increases in the training (**Figure 5B**) and validation (**Figure 5I**) sets. **Figures 5C, J** shown 20 TIPRGPI genes on the high and low TIPRGPI groups. The group with lower TIPRGPI exhibited a lower mortality rate compared to the high TIPRGPI group across the training (**Figures 5D, E**) and validation (**Figures 5K, L**) datasets. Subsequently, Kaplan-Meier analysis revealed statistically significant differences in overall survival probability between the high- and low- TIPRGPI groups across the training (**Figure 5F**) and validation (**Figure 5M**) datasets. Further, we used a time-dependent receiver operating characteristic curve analysis to assess the precision of the TIPRGPI signature. On the TCGA-SKCM training dataset, the area under the ROC curve (AUC) was 0.728, 0.744, 0.714 in 1-year, 2-year, and 3-year survival, respectively (**Figure 5G**). On the GSE65904 validation dataset, the AUC was 0.621, 0.668, 0.686 in 1-, 2-, and 3-year survival, respectively (**Figure 5N**). A stratified analysis demonstrated that TIPRGPI has added predictive value within subgroups categorized by age, gender, and stage (**Supplementary Figure 4**).

### Establishment of the prognostic nomogram

**Figure 6:**
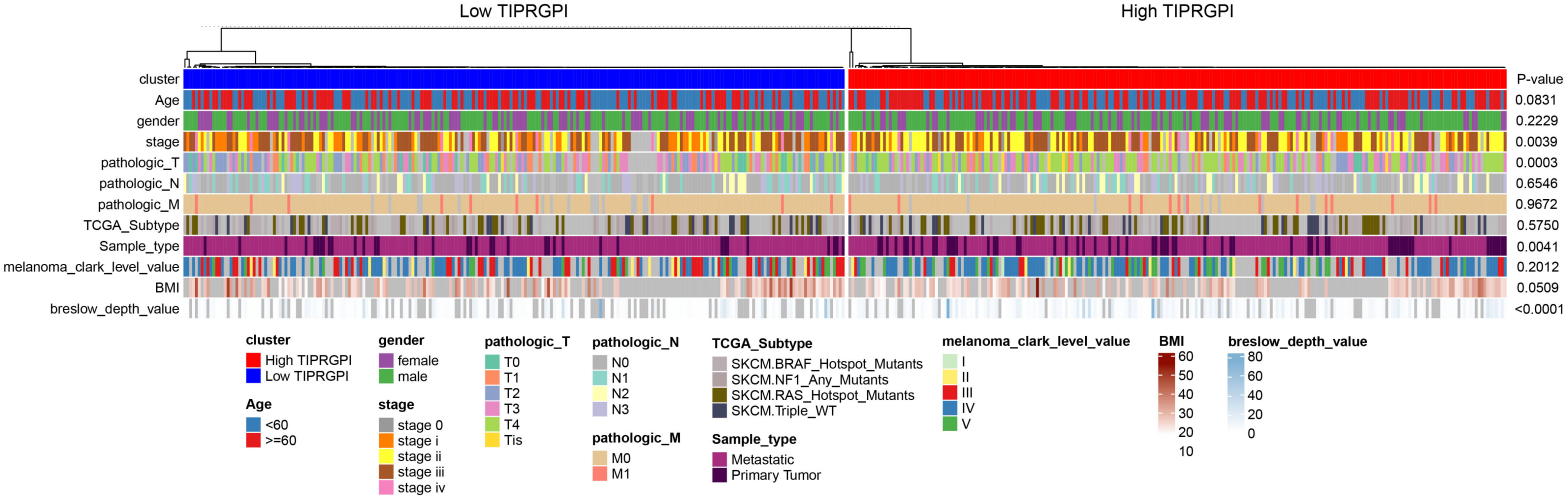
Differences in the distribution of various clinical factors in high- and low-TIPRGPI groups. There were significant differences in stage, T-stage, primary/metastasis, and Breslow depth.

**Figure 7:**
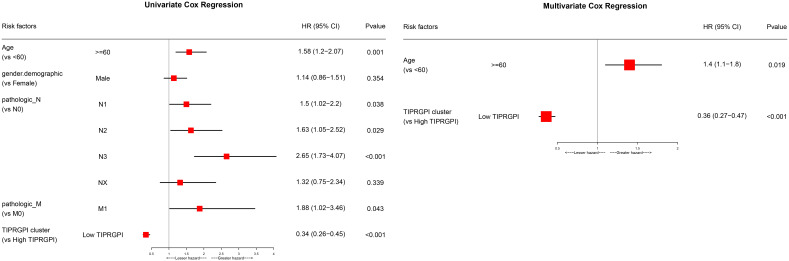
Univariate and multivariate analyses of the clinical traits and TIPRGPI for the OS in melanoma.

**Figure 8:**
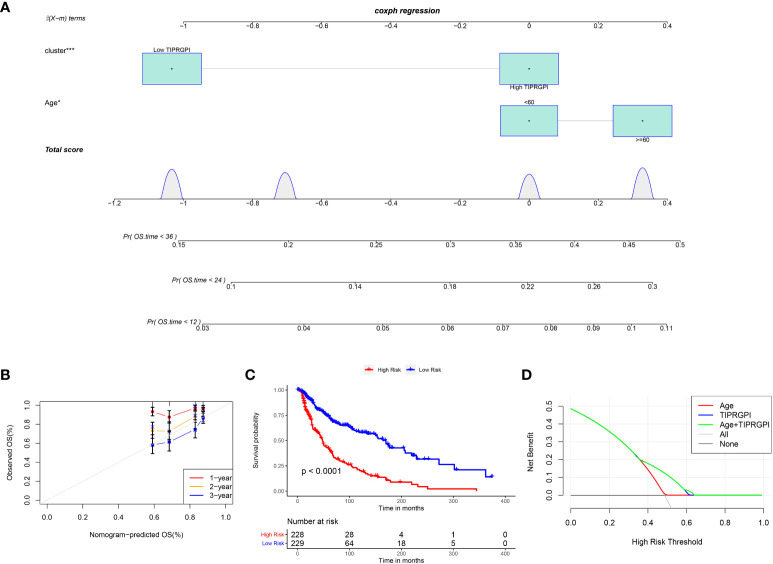
Evaluation of the TIPRGPI-integrated nomogram in melanoma. **(A)** Nomogram for predicting the probability of 1-, 2-, and 3-year OS. **(B)** The calibration plots of the nomogram predicting the probability of 1-, 2-, and 3-year OS. **(C)** Kaplan-Meier survival analysis of the age-TIPRGPI integrated nomogram for OS. **(D)** Decision curves showing the comparison of net benefits of the age, TIPRGPI, and age-TIPRGPI integrated for 2-year OS.

**Figure 6**; **Supplementary Figure 5** showed the differences in TIPRGPI scores between different clinical subgroups, as evidenced by significant differences in TIPRGPI scores between stage, T stage, metastatic, Clark level, and Breslow depth subgroups. To further determine whether the TIPRGPI predictive model served as an independent prognostic indicator in melanoma, both univariate and multivariate analyses were conducted. The HR of the TIPRGPI risk level was 0.34 (95%CI: 0.26-0.45) and 0.36 (95%CI: 0.27-0.47) in the univariate and multivariate analysis, respectively (**Figure 7**). Multivariate analysis shown TIPRGPI and age were independent prognostic factors in melanoma. To provide a quantitative instrument for physicians, a nomogram was built by age and TIPRGPI (**Figure 8A**). The calibration plot, which compares observed and predicted rates of 1-, 2- and 3-year overall survival, demonstrates the nomogram’s optimal consistency on OS in 2-year (**Figure 8B**). The survival curves of the high and low TIPRGPI groups in the composite model exhibit significant differences after incorporating the age variables (**Figure 8C**). **Figure 8D** showed TIPRGPI-age integrated nomogram achieved a better net benefit than TIPRGPI nomogram and age nomogram in predicting melanoma OS.

### Potential molecular mechanisms of TIPRGPI

**Figure 9:**
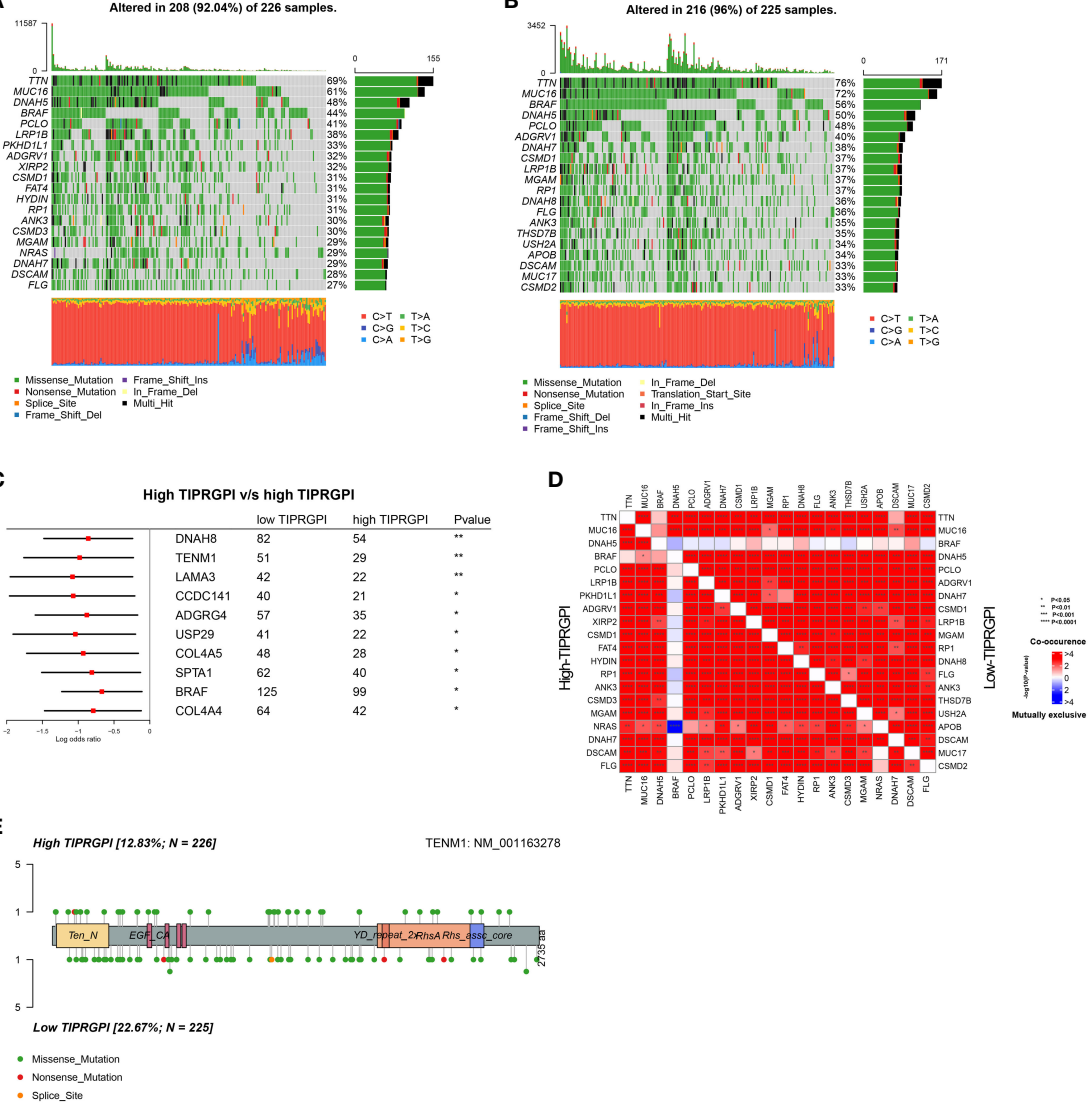
Genetic variations of the high- and low-TIPRGPI groups. **(A, B)** Waterfall plots showing the mutation landscapes of the high- **(A)** and low-TIPRGPI **(B)** groups, TTN and MUC16 mutations were obtained; **(C)** Forest plot showing significantly different mutated genes between high- and low-TIPRGPI groups; **(D)** The coincident and exclusive associations across the top mutated genes in high- and low-TIPRGPI groups; **(E)** Lollipop plot indicating the distribution of mutation spots in the high- and low-TIPRGPI groups.

We downloaded the available somatic mutation profiles and analyzed the mutational landscape of high- and low- TIPRGPI patients from the TCGA-SKCM dataset to explore the potential mechanisms underlying the risk level defined by TIPRGPI in melanoma. The top 20 genes with mutations were presented in two distinct groups. TTN (69%) and MUC16 (61%) exhibited the greatest frequency of mutation in both high- and low-TIPRGPI groups (**Figures 9A, B**). The significant differentially mutated genes between the high- and low- TIPRGPI groups were detected by Fisher’s exact test (**Figure 9C**), and the TENM1 were found with a much higher mutation rate in the high-TIPRGPI group compared with the low-TIPRGPI group (P<0.01). The mutation information summary with statistical calculations is presented in **Supplementary Table 4**. Meanwhile, the co-occurrence and exclusive associations of the top 20 mutated genes from the high- and low-TIPRGPI groups were also analyzed, where blue represents co-occurrence and red represents mutual exclusion (**Figure 9D**). A Lollipop plot has been used to show the different mutation sites of TENM1 (**Figure 9E**).

**Figure 10:**
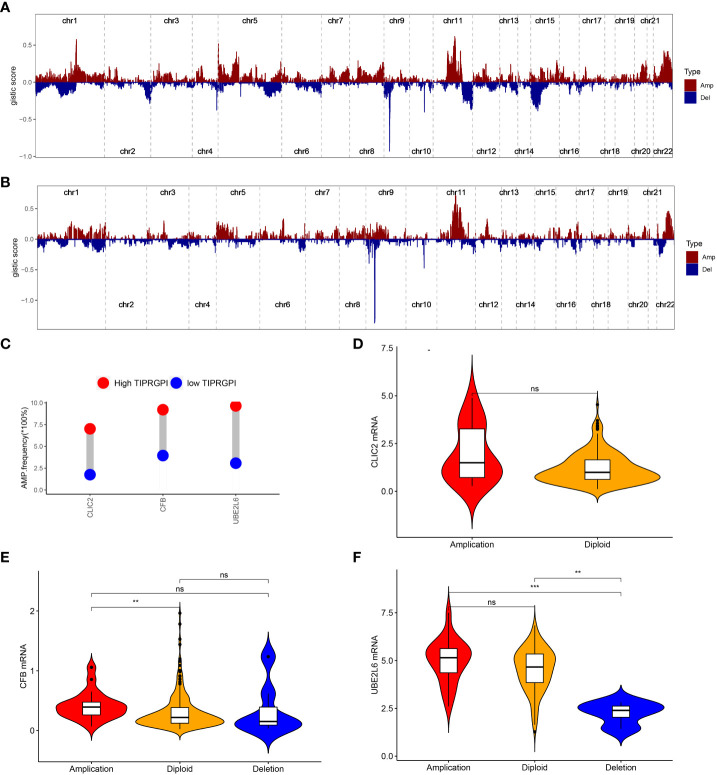
The distribution of CNV features across all chromosomes for the high- **(A)** and low- **(B)** TIPRGPI groups; **(C)** CLIC2, CFB and UBE2L6 showed significant differences at the CNV level; **(D)** Violin plots indicating the positive correlation of gene expression and copy number of CLIC2 in the high-TIPRGPI group; **(E)** Violin plots indicating the positive correlation of gene expression and copy number of CFB in the high- TIPRGPI group; **(F)** Violin plots indicating the positive correlation of gene expression and copy number of UBE2L6 in the high-TIPRGPI group.

The CNV alteration landscapes of the high- and low-TIPRGPI groups were generated after removing the germline features (**Figures 10A, B**). CLIC2, CFB and UBE2L6 were three genes that showed the positive correlations of gene expression in the high-TIPRGPI group (**Figures 10C–F**).

**Figure 11:**
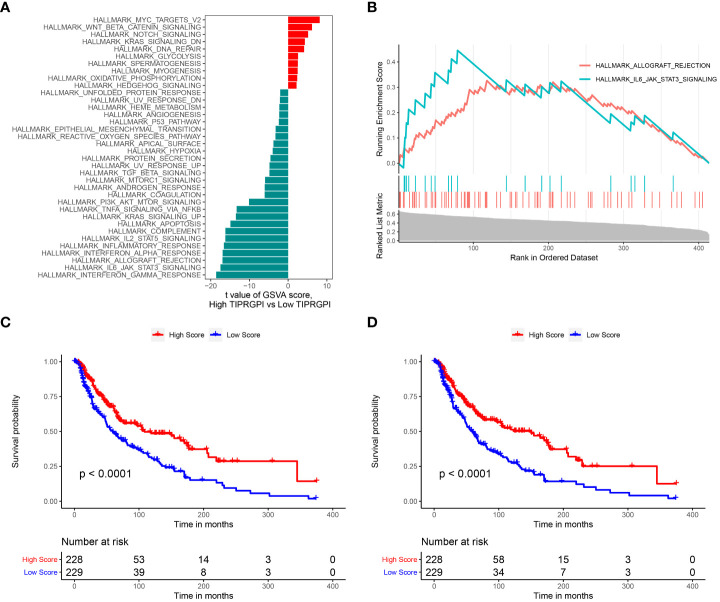
Determination of the distinct hallmark pathways of the high- and low TIPRGPI groups. **(A)** Differences in cancer hallmark pathway activities between the between the high- and low-TIPRGPI groups as assessed by GSVA; **(B)** The GSEA results for the 2 overlapping upregulated hallmark pathways in terms of the TIPRGPI groups; **(C)** Kaplan-Meier survival plots showing the significant correlations between the OS and GSVA scores of HALLMARK_ALLOGRAFT_REJECTION; **(D)** Kaplan-Meier survival plots showing the significant correlations between the OS and GSVA scores of HALLMARK_IL6_JAK_STAT3_SIGNALING.

We performed GSVA in high- and low-TIPRGPI groups. According to the predefined cutoff, 36 hallmark pathways were significantly increased in the high-TIPRGPI group compared to the low-TIPRGPI group (**Figure 11A**). GSEA confirmed that 2 of these genes were up-regulated in the high-TIPRGPI group of patients (**Figure 11B**). Kaplan-Meier survival analysis was utilized to assess the prognostic significance of the elevated hallmark pathways. Between the high- and low- TIPRGPI groups for these pathways, varying OS probabilities were noted (**Figures 11C, D**). HALLMARK_ALLOGRAFT_REJECTION is a pathway that is linked to organ transplant rejection, patients who underwent immunosuppression at the time of transplantation rejection displayed a better response to anti-PD1 therapy (18). The HALLMARK_IL6_JAK_STAT3_SIGNALING can lead to tumor cell proliferation, invasion, and metastasis (19).

### TIPRGPI was associated with melanoma TME

**Figure 12:**
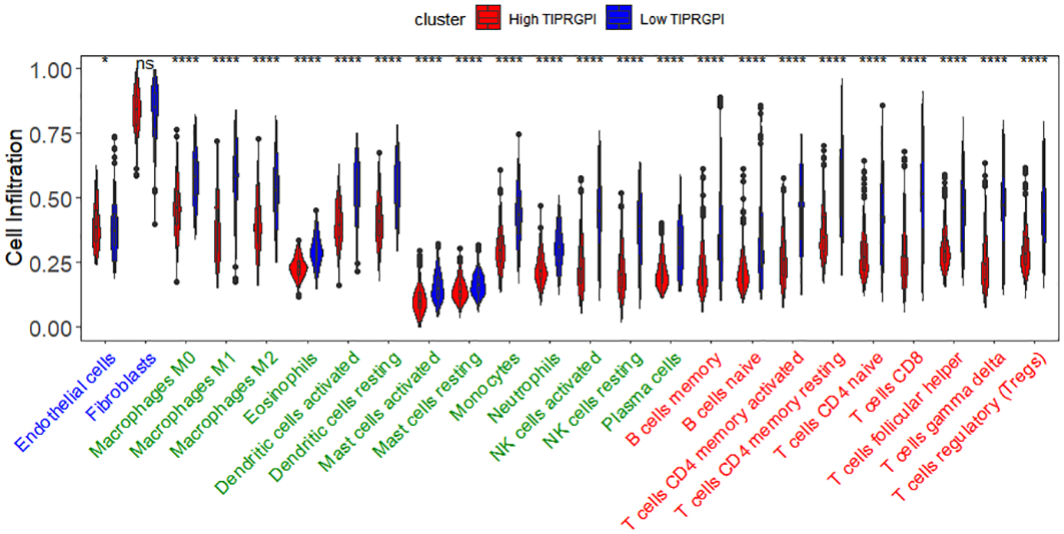
The differences of 23 TME cells infiltration between high- and low- TIPRGPI groups.

**Figure 12** illustrates the variance in infiltration between the high and low TIPRGPI groups across 24 cell types. It is shown that there is a significant difference in infiltration between the 23 cell types except fibroblasts. Then, we investigated the potential correlation between TIPRGPI and the TME cell infiltration. The significant correlation between the TIPRGPI score and infiltration scores of 23 TME cells, except for endothelial cells, which show a negative correlation (**Supplementary Figure 6**). The results of the one-way Cox regression demonstrate significant correlation between infiltration scores of 21 cells and survival, except for resting mast cells, endothelial cells, and fibroblasts. The 21 cells present in survival analysis were the most crucial factors in the correlation between TIPRGPI and TME cells (**Supplementary Figure 7**). TIPRGPI constituent genes correlation with TME-associated cells between high- and low- TIPRGPI groups and revealed that the infiltration of most TME cell types including naive B cells, memory B cells, memory CD4 T cells, naïve CD4 T cells, CD8 T cells, follicular helper T cells, gamma delta T cells, and regulatory T cells (P<0.05) were significantly associated with TIPRGPI (**Supplementary Figure 8**). There was a significant difference in the immune cell recruitment、immune suppression、innate immunity、adaptive immunity、antigen presentation and processing、cytotoxicity/killing of cancer cells、inflammation gene sets between the high and low TIPRGPI groups (**Supplementary Figure 9**).

### TIPRGPI may be a sensitive predictor of immunotherapy

**Figure 13:**
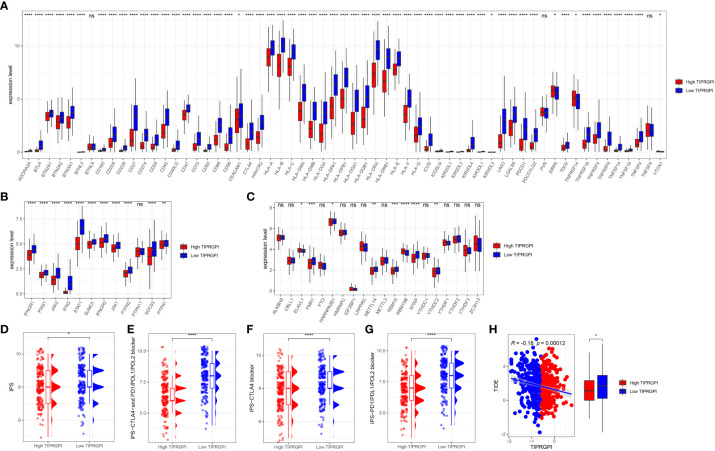
Application of the TIPRGPI model for immunotherapy prediction in melanoma. **(A)** The high- and low-TIPRGPI groups were significantly different in 58 out of 61 immune checkpoints; **(B)** The high- and low-TIPRGPI groups were significantly different in 11 of the 12 interferon-γ pathway marker genes; **(C)** The high- and low-TIPRGPI groups were significantly different in 8 of the 20 m6A regulators; **(D–G)** The relationship between TIPRGPI and IPS, significant difference in all 4 subgroups; **(H)** TIDE correlated with TIPRGPI, which showed a significant negative correlation.

**Figure 14:**
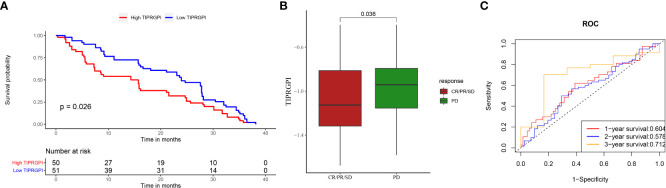
Applying TIPRGPI to assess immunotherapy prognosis in the GSE91061 melanoma immunotherapy dataset. **(A)** Significant difference in survival between high- and low-TIPRGPI groups in the GSE91061 dataset; **(B)** Significant difference in TIPRGPI scores between immunotherapy CR, PR, SD and PD groups; **(C)** ROC curve.

To evaluate the potential of TIPRGPI to predict how melanoma patients respond to immunotherapy, we first determined the expression of 61 immunomodulators in high- and low- TIPRGPI groups. **Figure 13A** showed that the high- and low- TIPRGPI groups were significantly different in 58 of 61 immune checkpoints. **Figure 13B** shows that the high- and low- TIPRGPI groups were significantly different in 11 of 12 interferon-γ pathway marker genes. **Figure 13C** shows that the high- and low- TIPRGPI groups were significantly different in 8 of 20 m6A regulators. **Figures 13D–G** show the differences between the high- and low- TIPRGPI groups in the IPS subgroups, as shown by the significant differences in all 4 subgroups. **Figure 13H** shows the TIDE-TIPRGPI correlation, which is significantly negatively correlated, with lower TIDE scores in the high-TIPRGPI group. We also predict the immunotherapeutic efficacy for immune checkpoint blockade in GSE91061, the high-TIPRGPI group had a better OS, and the AUC was 0.604,0.578,0.712 in 1-, 2-, and 3-year survival, respectively. (**Figure 14**).

### Core target identification and candidate molecules prediction

To identify the core target in relation to TIPRGPI, a PPI network was constructed using the STRING database (confidence score>0.4). There were 87 differential genes between the high- and low- TIPRGP groups (**Supplementary Table 5**). The PPI interaction network constructed using the 87 differential genes, in which the protein tyrosine phosphatase receptor type C (PTPRC), which has the most connections to the other nodes, is shown as a core factor in red in the center of the figure (**Supplementary Figure 10**). Thus, PTPRC was considered as core target. PTPRC is a member of the protein tyrosine phosphatase (PTP) family, which is mainly involved in the regulation of cell growth, differentiation, mitosis, and oncogenic transformation.

**Figure 15:**
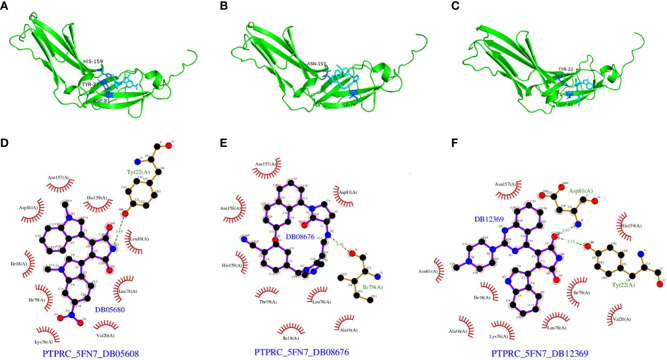
Docking conformation and interaction force analysis. **(A)** Pymol 3D structures and binding modes showing the formed hydrogen bonds between the predicted pocket of PTPRC and DB08676 **(A)**, DB05608 **(B)** and DB12369 **(C)**; Ligplus interaction force analysis showing hydrogen bonds formed by DB08676 **(D)**, DB05608 **(E)** and DB12369 **(F)** with amino acid residues of proteins.

We also analyzed potential molecules that might interact with PTPRC through molecular docking. The corresponding compound structure information was downloaded from the DrugBank database and screened according to Lipinski’s rule (hydrogen bond acceptor ≤10, hydrogen bond donor ≤ 5, rotatable bond≤ 10, logarithmic value of lipid-water partition coefficient≤ 5, molecular weight of 180-480, and polar surface area≤ 140), and finally 5462 small molecule compounds were obtained. The spatial structure information of the key gene-encoded proteins was searched in the PDB database to find the corresponding data of PTPRC, and the corresponding PDB (5FN7) file were downloaded. Docking with the small molecule compounds was performed using Autodock-Vina, and interaction force analysis was performed using Pymol and Ligplus, and the top 10 small molecule compounds that had the best scores for binding to the PTPRC are as showed in **Table 4**. **Figure 15** showed the docking conformation and interaction of PTPRC with DB08676, DB05608, and DB12369.

**Table 4:** Top 10 small molecule compounds had the best scores for binding to the PTPRC.

DrugBank ID	Hydrogen Acceptors	Hydrogen Donors	Rotatable Bonds	LogP	Molecular Weight	TPSA	Affinity (kcal/mol)
DB08676	5	1	0	2.5	453.5	83.2	-8.2
DB05608	4	1	2	2.6	400.4	102	-8
DB12369	6	2	3	2.6	438.5	94.2	-7.9
DB14773	8	2	3	3.7	478.4	89.1	-7.9
DB15345	8	1	5	1.1	451.5	79.8	-7.9
DB12134	7	1	5	0.1	448.5	90.4	-7.8
DB01395	3	0	0	3.5	366.5	43.4	-7.7
DB15442	7	2	3	2.6	446.5	91.2	-7.7
DB12368	7	1	4	3.2	431.4	77	-7.7
DB12690	9	1	3	3.6	445.4	75.5	-7.7

## Discussion

The tumor immune-microenvironment is intricately linked to the tumorigenesis and the progression of cancer. The clinical success with ICB in melanoma has confirmed the therapeutic impact of re-invigorating the immune system to effectively target melanoma. However, even with combination ICB in optimal scenarios, approximately half of patients do not experience durable benefit. Therefore, a effective prediction model for melanoma immunotherapy is important for the clinical management of this disease.

The TIP genes guided strategy, utilizing multiple statistical approaches, has resulted in the development of a novel and effective immune-relevant predictive model-TIPRGPI, which showed promising results for prognosis and immunotherapy in certain cancer (16). For the first time, we conducted WGCNA to uncover the specific gene expression pattern associated with TIP score in melanoma. We identified a melanoma TIPRGPI module which generated a 22-gene signature for predicting melanoma prognosis, and our results suggest that lower TIPRGPI indicates better prognosis.

In this study, there are 20 genes and their coefficients in the TIPRGPI model, offer valuable insights into the distribution of immune factors and potential immunotherapeutic biomarkers associated with melanoma. In 2018, a review concluded that Notch3 signaling may play an important role in oncogenesis, tumor maintenance, and resistance to chemotherapy (20). In 2022, Serge Fuchs and colleagues reported that targeting PARP11 may avert immunosuppression and improve CAR-T therapy in solid tumors. In 2023, Yan Cao and colleagues reported that TUBB4A was enriched in pathways related to melanoma, and *in vitro* experiments indicated that TUBB4A promoted proliferation and migration of melanoma cells A375 and B16-F10 (21). In the same year, Hui-Min He and colleagues performed *in vitro* experiments and found that TRIM69 maybe a potential sensitizer to PD-1 blockers (22). In our study, the coefficient of TRIM69 was negative, implying that high TRIM69 expression, low TIPRGPI scores and good prognosis, consistent with the results of the *in vitro* study. The same negative coefficient was found for CLIC2, a dimorphic protein present in both soluble and membrane fractions. CLIC2 was found to be expressed at higher levels in benign tumors than in malignant tumors, most likely preventing tumor cell invasion into surrounding tissues. CLIC2 was also expressed in vascular endothelial cells of normal tissues and maintains their intercellular adhesive junctions, presumably suppressing hematogenous metastasis of malignant tumor cells (23). A significant difference was identified in the distribution of immune factors between the high- and low-TIPRGPI groups, suggesting that TIPRGPI may assist in the risk stratification of melanoma patients, providing further evidence of the heterogeneity of melanoma and its potential impact on treatment outcomes.

Additionally, key gene modules associated with TIPRGPI were identified. Among these modules, the protein PTPRC was identified as a core target. The identification of PTPRC as a core target suggests its potential role in the development and progression of melanoma. PTPRC, belongs to the PTP family, also known as CD45, is a transmembrane glycoprotein, expressed on almost all hematopoietic cells except for mature erythrocytes, and is an essential regulator of T and B cell antigen receptor-mediated activation. Imbalance between the activity of protein tyrosine kinase and phosphatase, due to CD45 and other factors, can lead to immunodeficiency, autoimmunity, or malignancy (24). PTPRC participates in a range of cellular processes, including cell growth, differentiation, and oncogenic transformation. Our previous study demonstrated that PTP1B promotes the progression of melanoma cells *in vitro* and *in vivo* (25). In 2021, a pan-cancer study highlighted the significance of PARP1 alterations as cancer predictive biomarkers for immunotherapy, and its expression levels were correlated with the status of immunotherapy-associated signatures in several tumors (26). Further bioinformatics investigations have indicated the pivotal function of PTPRC in melanoma. In 2021, Thomas F Gajewski and colleagues reported that up to 90% of PTPRC+ cells produced CXCL10 transcripts, which played a critical role in recruiting effector CD8+ T cells to the tumor site (27). In 2022, Xiaobo Xia and colleagues reported that the OIP5-AS1-PTPRC/IL7R/CD69 axis in ceRNA as a clinical prognostic model (28).

The study employed molecular docking analysis to identify potential small molecule compounds that may interact with PTPRC. This approach allows for the identification of potential therapeutic targets and the development of novel treatment strategies. The screening of small molecule compounds based on specific criteria, such as Lipinski’s rule, ensures the selection of compounds with favorable drug-like properties.

The TIPRGPI model demonstrated significant differences in OS between the high and low groups in both the TCGA training set, the GSE65904 validation set, and the GSE91061 immunotherapy dataset, with no intersection of survival curves among subgroups. Our model also demonstrates a higher AUC than most previous studies aimed at developing effective risk classifiers for melanoma. For instance, in 2022, Song et al. reported a necroptosis-related gene signature in cutaneous melanoma that achieved a 2-year AUC of 0.700 in the training dataset and 0.634 in the validation dataset (29). In contrast, TIPRGPI model achieved a 2-year AUC of 0.744 in the training dataset and 0.668 in the validation dataset. Previously, our team presented a G protein-coupled receptor-based prediction model that predicted 1-, 3-, and 5-year overall survival with AUCs ranging from 0.672 to 0.703 (30). Chuang et al. reported an AUC of 0.623 on breast cancer outcome prediction (31). In 2023, a pan-cancer immunotherapy response study built a predictive model based on endothelial senescence and showed that the machine learning algorithm “KKNN” had the highest AUC for response at 0.75 in the anti-PD-1/PD-L1 treated melanoma cohort (PUCH SKCM, 2021), while in other melanoma immunotherapy cohorts the AUC decreased to around 0.5, and the AUC for OS was even lower (32). Generally, the AUC of existing OS prediction models ranges from 0.6 to 0.7, and our model demonstrates similar predictive performance compared to the existing models. Particularly, our TIPRGPI predicts the immunotherapeutic efficacy of immune checkpoint blockade in GSE91061, with an AUC of 0.712 for the 3-year survival rate, which is considered relatively high within existing prognostic models for melanoma.

Despite the improved AUC for OS of our model compared to previous models, we emphasize that it still needs improvement. The model demonstrates strong predictive capability on the training set, with all AUC values exceeding 0.7. Moreover, on the validation set, the AUC values for 2 and 3 years are both greater than 0.65, indicating a certain level of predictive ability. However, further improvement is still needed in predicting 1-year survival.

Although the recently studies reported better predictive validity of their models compared to ours, these were studies based on single-cell sequencing data (32) or imaging data (33), whereas ours was based on bulk-seq. It is impossible to compare predictive validity between models based on different types of data. Meanwhile, these recently emerged technologies are not without flaws. Single-cell sequencing requires a trade-off between breadth (sequencing more cells) and depth (sequencing more transcripts per cell) and is subject to experimental cost pressures (34). Certain studies have introduced models employing deep learning (DL), a branch primarily applied in image recognition. Within the realm of medical image analysis, the task of label annotation poses persistent challenges during the development of DL models. The inherent opacity of DL algorithms often renders their inner workings inscrutable, colloquially termed as a “black box.” In contrast, our models are primarily constructed using clustering trees and logistic regression. This approach ensures a higher level of interpretability and transparency, avoiding “black box”.

Our study presents several advances over previous literature (35). Firstly, a series of related marker genes were clearly identified. Furthermore, the model constructed in this study has significance in predicting immunotherapy response and screening therapeutic drug candidates in addition to its prognostic significance.

This study is limited by its bioinformatics approach. Further research, such as flow cytometry and immunohistochemistry, is warranted to validate these findings and explore their clinical implication. Second, to enhance the robustness of our conclusions, we recommend utilizing an internal cohort that includes gene expression data, survival data, and immunotherapy response data to further assess the performance of the TIPRGPI in melanoma. The findings from this study contribute to our understanding of the molecular mechanisms underlying melanoma and provide insights into potential biomarkers and therapeutic targets. The identification of specific clinical factors, gene modules, and immune markers associated with TIPRGPI may aid in the development of personalized treatment strategies for melanoma patients.

## Conclusion

In conclusion, melanoma patients with lower TIPRGPI indicate better prognosis and better immunotherapy response. As the TIPRGPI increases, infiltration of almost all types of immune cells (21 types except endothelial cells) decreases, resulting in a poorer prognosis and a greater likelihood of progression even after immunotherapy. The analysis of high- and low- TIPRGPI groups provides valuable information regarding the distribution of clinical factors, gene modules, and immune markers associated with melanoma.

## Data availability statement

The datasets presented in this study can be found in online repositories. The names of the repository/repositories and accession number(s) can be found in the article/**Supplementary Material**.

## Author contributions

SZ: Writing – original draft, Writing – review & editing. AH: Writing – original draft, Writing – review & editing. CC: Writing – original draft, Writing – review & editing. JG: Writing – original draft, Writing – review & editing. CW: Writing – original draft, Writing – review & editing. ZC: Writing – original draft, Writing – review & editing. JL: Writing – original draft, Writing – review & editing.

## Supplementary material

Supplementary Figure 1 Correlations between TIP score and immune score **(A)**, stromal score **(B)**, estimate score **(C)**, tumor purity **(D)**, activated CD4/CD8 **(E, F)** and PD-1/CTLA-4 **(G, H)**. Tumor purity was negatively correlated with TIP score and the rest were positively correlated.

Supplementary Figure 2 TIP score correlations with TMB and TIDE were all significant but not strong.

Supplementary Figure 3 GO and KEGG enrichment analysis. **(A)** GO enrichment analysis. **(B)** KEGG analysis.

Supplementary Figure 4 Survival curves for different clinical subgroups of high- and low- TIPRGPI groups. **(A)** age <60 years; **(B)**: age ≥60 years; **(C)** female; **(D)** male; **(E)** stage 0/I/II; **(F)**: stage III/IV.

Supplementary Figure 5 Analysis in TIPRGPI scores across clinical factors. **(A)** age; **(B)** gender; **(C)** stage; **(D)** T stage; **(E)** N stage; **(F)** M stage; **(G)** primary/metastasis; **(H)** Clark level; **(I)** BMI; **(J)** Breslow depth; **(K)**: TCGA molecular staging. (ns: p>0.05; *: p ≤ 0.05; **: p ≤ 0.01; ***: p ≤ 0.001; ****: p ≤ 0.0001).

Supplementary Figure 6 The correlation between the infiltration scores of the 23 different TME cells and the TIPRGPI score was found to be significantly correlated, except for endothelial cells, which were negatively correlated with the TIPRGPI score. (adaptive immunity in red, intrinsic immune cells in green, and stromal cells in blue).

Supplementary Figure 7 TIPRGPI cells most relevant to TME. **(A)** Venn diagram revealing 21 types of most relevant TME cells contributing to the risk stratification of melanoma patients by TIPRGPI; **(B)** The bar graphs show the number of cells in the differential analysis, correlation analysis and survival analysis.

Supplementary Figure 8 Cellular relevance of TIPRGPI constituent genes most relevant to TME. (ns: p>0.05; *: p ≤ 0.05; **: p ≤ 0.01; ***: p ≤ 0.001; ****: p ≤ 0.0001).

Supplementary Figure 9 Differences in expression of seven immune-related gene sets (immune cell recruitment, immune suppression, innate immunity, adaptive immunity, antigen presentation and processing, cytotoxicity/killing of cancer cells, inflammation) between high- and low-TIPRGPI groups. There were significantly different in enrichment in all 7 gene sets (ns: p>0.05; *: p ≤ 0.05; **: p ≤ 0.01; ***: p ≤ 0.001; ****: p ≤ 0.0001).

Supplementary Figure 10 The total number of differential genes between the high- and low-TIPRGP groups was 87, and the PPI interaction network constructed using the 87 differential genes, in which the protein PTPRC with the most connections to other nodes in red at the right center of the figure was used as a core factor.
