# Supplementary figures and images for: Predicting immunotherapy response in melanoma using a novel tumor immunological phenotype-related gene index
Source: Front Immunol. 2024 Mar 20;15:1343425. doi: 10.3389/fimmu.2024.1343425 (PMC10987686; doi:10.3389/fimmu.2024.1343425)

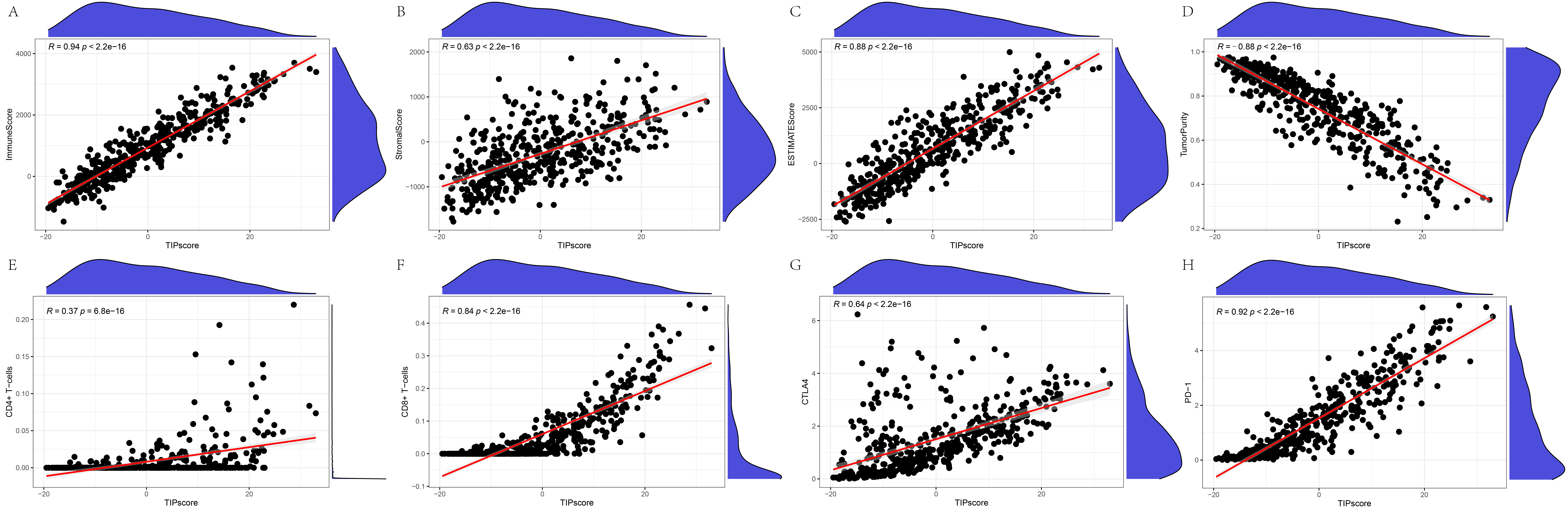

Supplement: Supplementary Figure 1 — Correlations between TIP score and immune score (A), stromal score (B), estimate score (C), tumor purity (D), activated CD4/CD8 (E, F) and PD-1/CTLA-4 (G, H). Tumor purity was negatively correlated with TIP score and the rest were positively correlated. [file Image_1.tif]

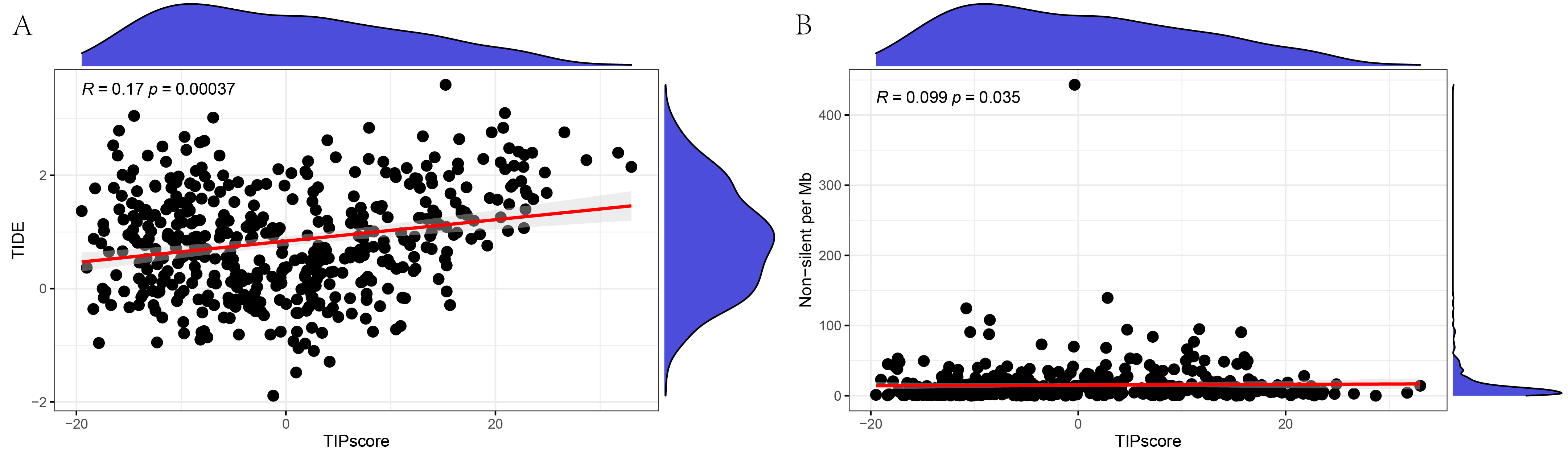

Supplement: Supplementary Figure 2 — TIP score correlations with TMB and TIDE were all significant but not strong. [file Image_2.tif]

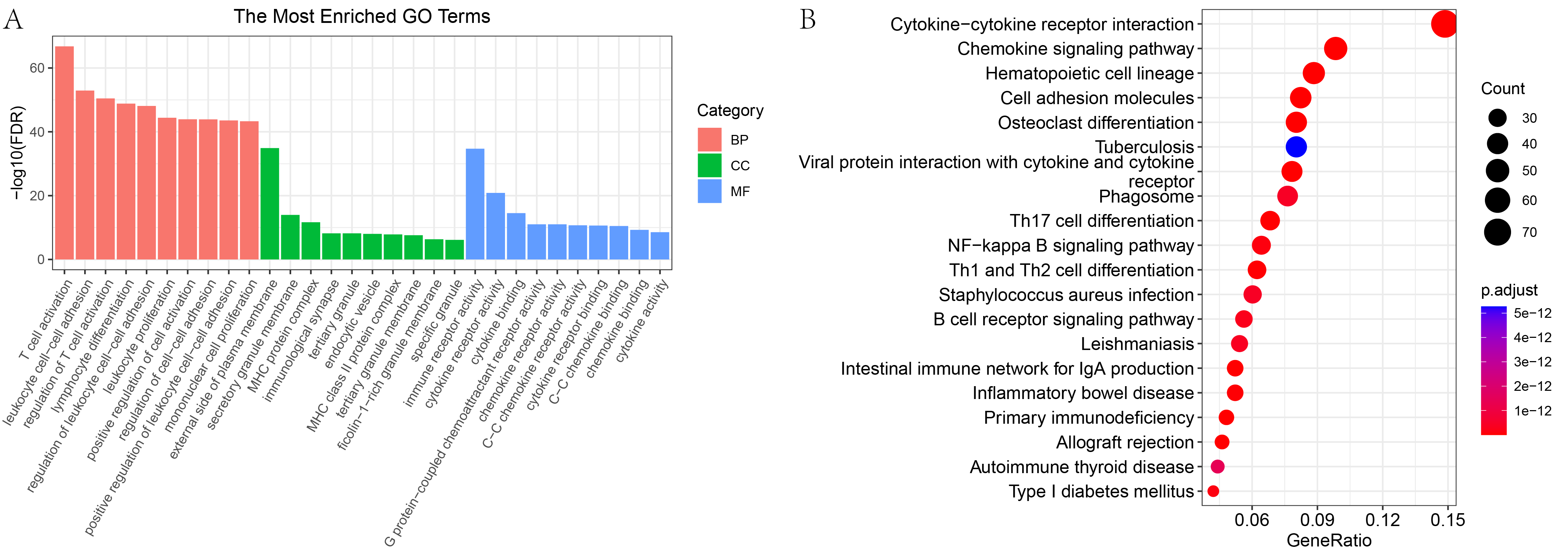

Supplement: Supplementary Figure 3 — GO and KEGG enrichment analysis. (A) GO enrichment analysis. (B) KEGG analysis. [file Image_3.tif]

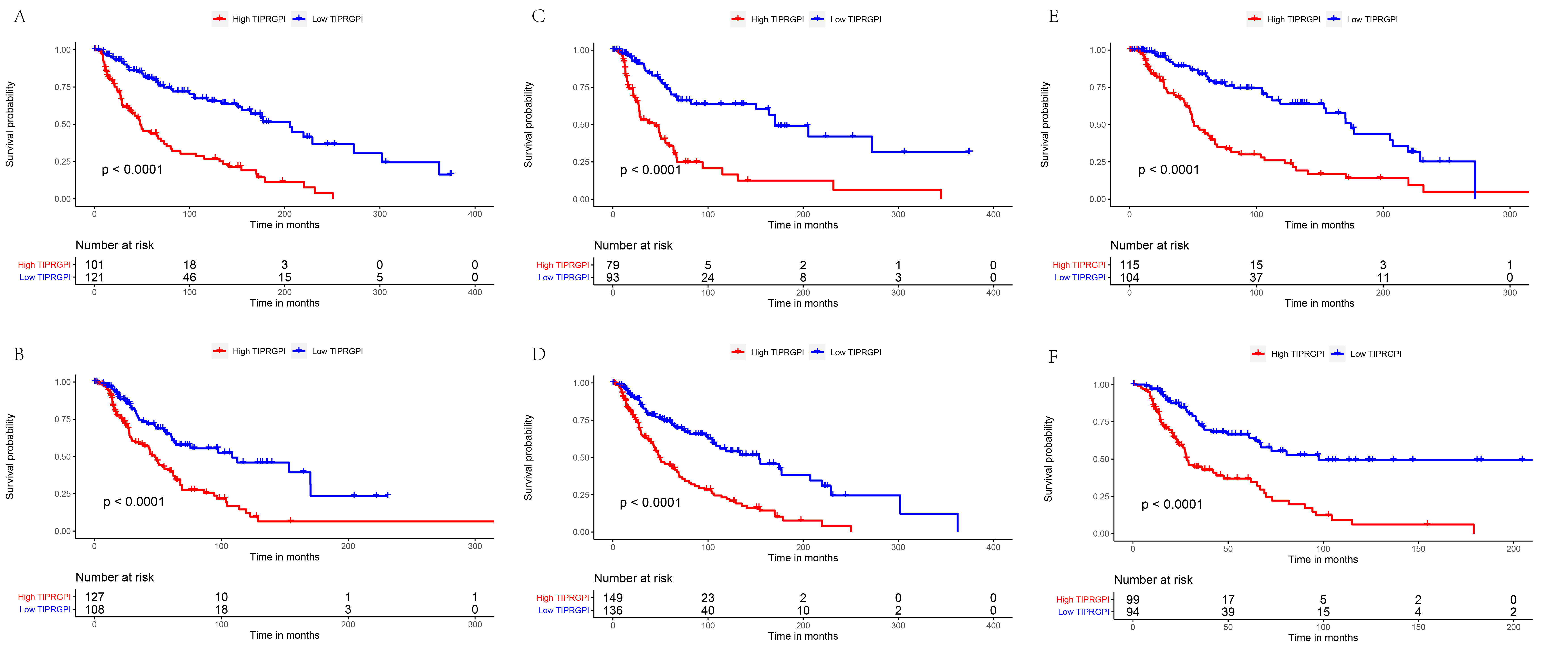

Supplement: Supplementary Figure 4 — Survival curves for different clinical subgroups of high- and low- TIPRGPI groups. (A) age <60 years; (B): age ≥60 years; (C) female; (D) male; (E) stage 0/I/II; (F): stage III/IV. [file Image_4.tif]

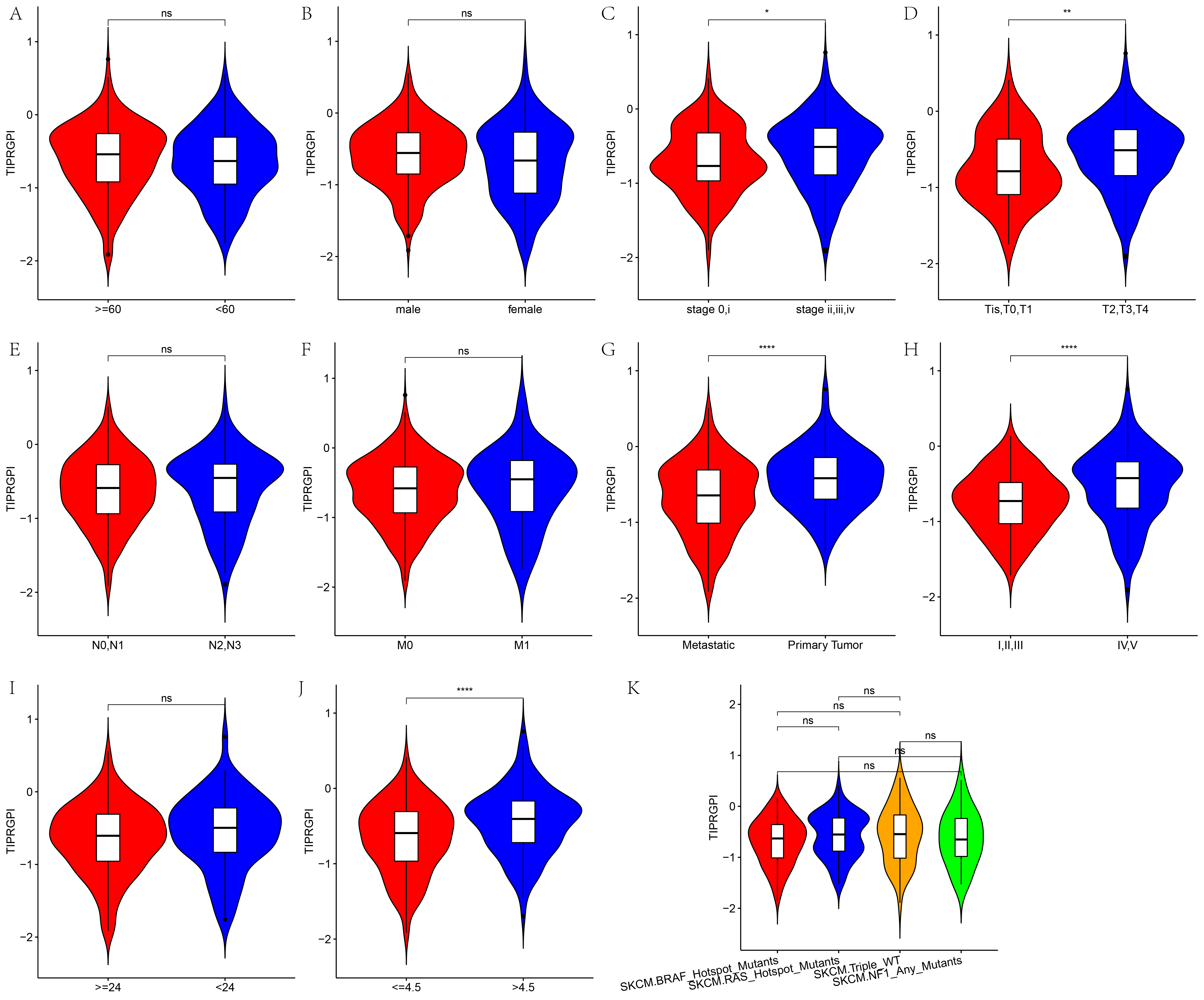

Supplement: Supplementary Figure 5 — Analysis in TIPRGPI scores across clinical factors. (A) age; (B) gender; (C) stage; (D) T stage; (E) N stage; (F) M stage; (G) primary/metastasis; (H) Clark level; (I) BMI; (J) Breslow depth; (K): TCGA molecular staging. (ns: p>0.05; *: p ≤ 0.05; **: p ≤ 0.01; ***: p ≤ 0.001; ****: p ≤ 0.0001). [file Image_5.tif]

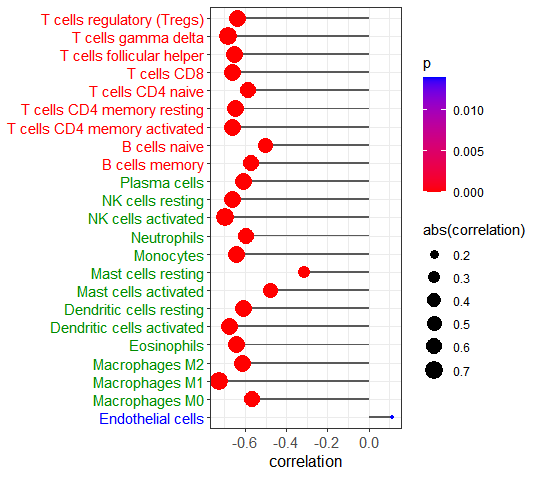

Supplement: Supplementary Figure 6 — The correlation between the infiltration scores of the 23 different TME cells and the TIPRGPI score was found to be significantly correlated, except for endothelial cells, which were negatively correlated with the TIPRGPI score. (adaptive immunity in red, intrinsic immune cells in green, and stromal cells in blue). [file Image_6.tiff]

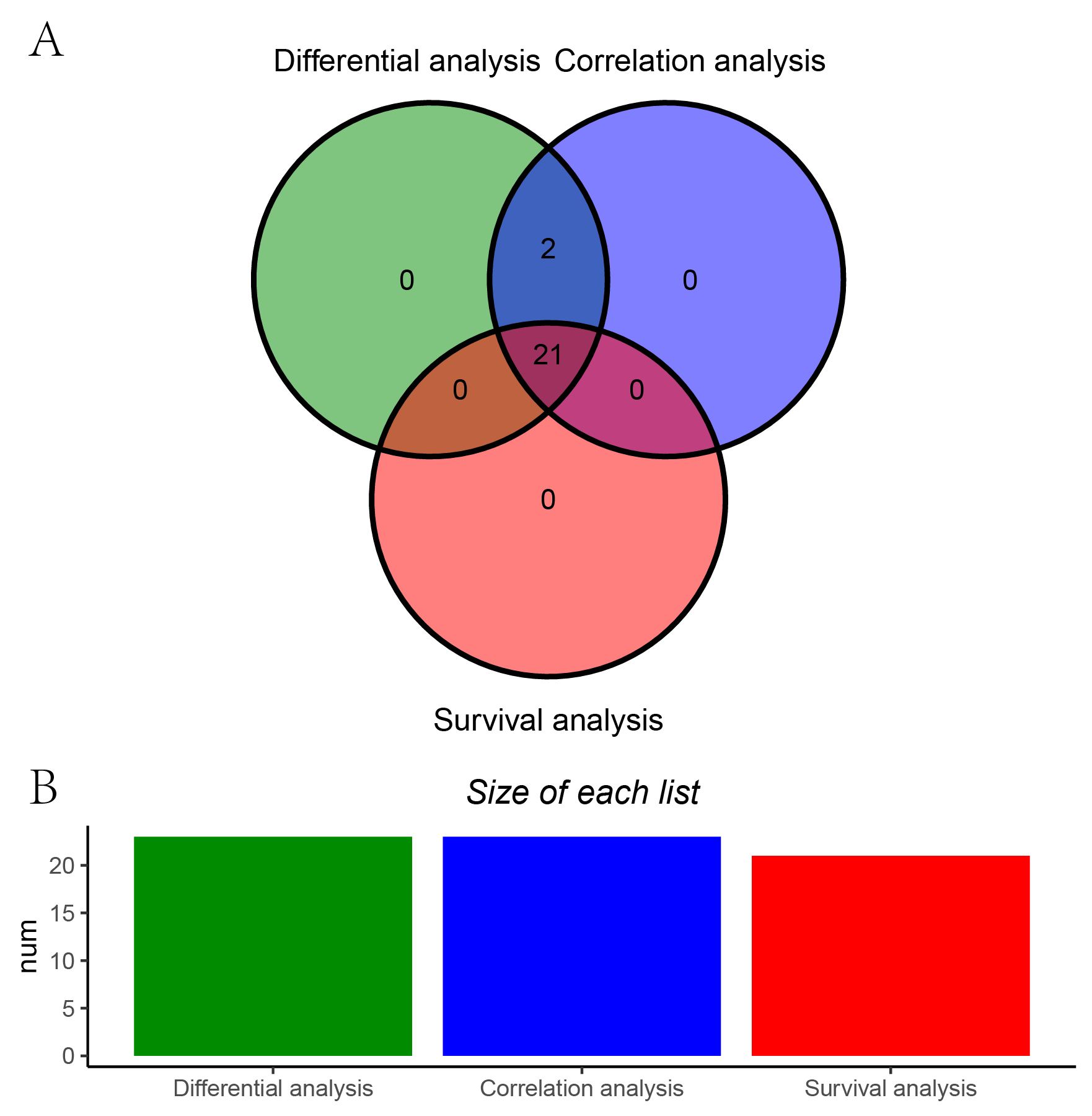

Supplement: Supplementary Figure 7 — TIPRGPI cells most relevant to TME. (A) Venn diagram revealing 21 types of most relevant TME cells contributing to the risk stratification of melanoma patients by TIPRGPI; (B) The bar graphs show the number of cells in the differential analysis, correlation analysis and survival analysis. [file Image_7.tif]

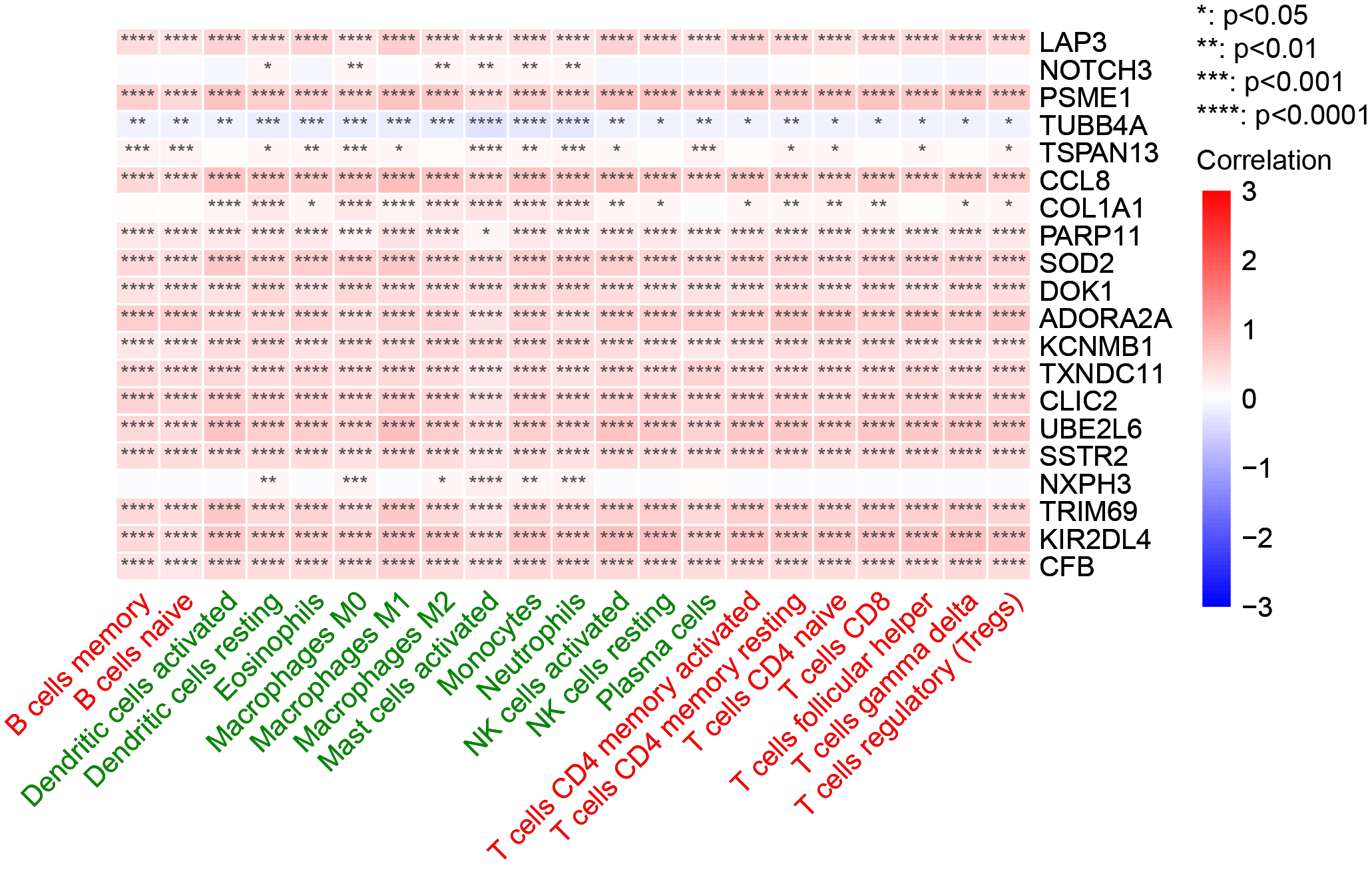

Supplement: Supplementary Figure 8 — Cellular relevance of TIPRGPI constituent genes most relevant to TME. (ns: p>0.05; *: p ≤ 0.05; **: p ≤ 0.01; ***: p ≤ 0.001; ****: p ≤ 0.0001). [file Image_8.tif]

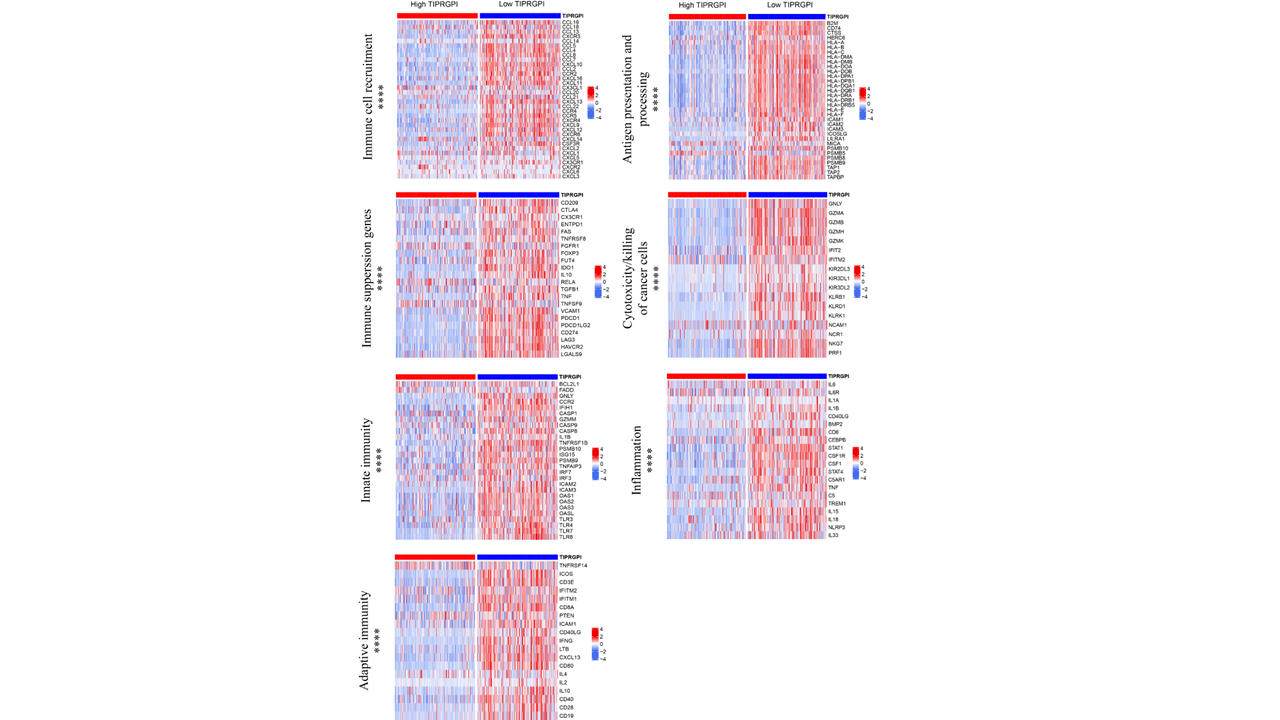

Supplement: Supplementary Figure 9 — Differences in expression of seven immune-related gene sets (immune cell recruitment, immune suppression, innate immunity, adaptive immunity, antigen presentation and processing, cytotoxicity/killing of cancer cells, inflammation) between high- and low-TIPRGPI groups. There were significantly different in enrichment in all 7 gene sets (ns: p>0.05; *: p ≤ 0.05; **: p ≤ 0.01; ***: p ≤ 0.001; ****: p ≤ 0.0001). [file Image_9.tif]

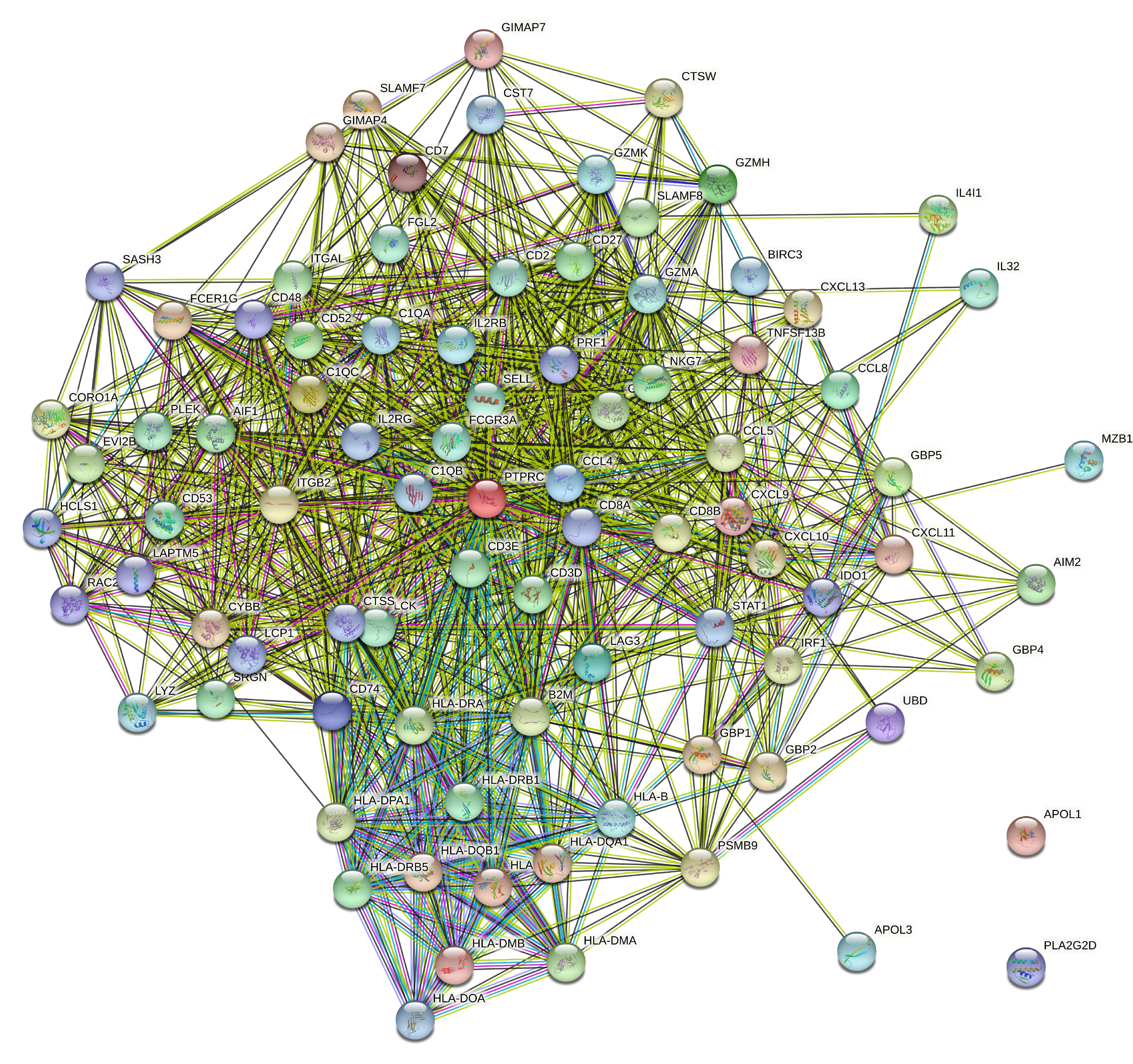

Supplement: Supplementary Figure 10 — The total number of differential genes between the high- and low-TIPRGP groups was 87, and the PPI interaction network constructed using the 87 differential genes, in which the protein PTPRC with the most connections to other nodes in red at the right center of the figure was used as a core factor. [file Image_10.tif]
