# Supplementary material for: Predicting immunotherapy response in melanoma using a novel tumor immunological phenotype-related gene index
Source: Front Immunol. 2024 Mar 20;15:1343425. doi: 10.3389/fimmu.2024.1343425 (PMC10987686; doi:10.3389/fimmu.2024.1343425)
Supplement: Supplementary file 11 [file DataSheet_1.zip › Data Sheet 1.DOCX]

TIPscore Sample TIP cluster

7.70497983260816 TCGA-EE-A2GJ-06A High TIPscore

10.5405536906989 TCGA-EE-A2GI-06A High TIPscore

-15.6749636587836 TCGA-WE-A8ZM-06A Low TIPscore

-12.8869641220441 TCGA-DA-A1IA-06A Low TIPscore

20.4954759519239 TCGA-D3-A51H-06A High TIPscore

-14.2326931214583 TCGA-FS-A1ZE-06A Low TIPscore

16.4322420815741 TCGA-D3-A8GB-06A High TIPscore

-18.3575331614362 TCGA-D3-A8GL-06A Low TIPscore

-10.4449228479309 TCGA-BF-A5EP-01A Low TIPscore

-10.9055563691239 TCGA-FS-A1ZS-06A Low TIPscore

2.85199221239692 TCGA-ER-A19D-06A High TIPscore

1.83760667757796 TCGA-D3-A2J6-06A High TIPscore

1.53692620890863 TCGA-EE-A3AF-06A High TIPscore

-7.33223957994954 TCGA-BF-AAP6-01A Low TIPscore

4.71721969358727 TCGA-D3-A8GM-06A High TIPscore

3.0603716273062 TCGA-D9-A3Z3-06A High TIPscore

2.99831087688872 TCGA-EE-A2MH-06A High TIPscore

11.1162817535009 TCGA-EE-A2GC-06A High TIPscore

0.634296639547045 TCGA-WE-A8K5-06A High TIPscore

-15.5103956463794 TCGA-EE-A17Z-06A Low TIPscore

-19.4958069555364 TCGA-ER-A19T-01A Low TIPscore

-4.68020859879694 TCGA-FS-A4FC-06A Low TIPscore

-2.73446734469805 TCGA-XV-A9W5-01A Low TIPscore

-12.5002471186757 TCGA-ER-A42H-01A Low TIPscore

-2.97320976701914 TCGA-EB-A44P-01A Low TIPscore

7.70323965551036 TCGA-FS-A1ZT-06A High TIPscore

-17.4992553010118 TCGA-GN-A8LK-06A Low TIPscore

14.6457109915568 TCGA-FR-A729-06A High TIPscore

12.8707624628999 TCGA-D3-A51J-06A High TIPscore

-5.61903494674712 TCGA-EB-A57M-01A Low TIPscore

6.59808065340653 TCGA-WE-A8ZN-06A High TIPscore

-0.497498332253195 TCGA-EE-A2MD-06A High TIPscore

7.43224866328241 TCGA-DA-A1I1-06A High TIPscore

7.04220909084495 TCGA-EB-A82B-01A High TIPscore

-16.434648022882 TCGA-FS-A4FB-06A Low TIPscore

-17.3695989128916 TCGA-EE-A3J8-06A Low TIPscore

20.5099567511014 TCGA-D3-A1Q7-06A High TIPscore

1.00181410792727 TCGA-GN-A267-06A High TIPscore

11.6723449522724 TCGA-GN-A266-06A High TIPscore

-7.49654839374966 TCGA-D3-A8GC-06A Low TIPscore

-12.7141925479618 TCGA-BF-AAOX-01A Low TIPscore

3.1848677257851 TCGA-D3-A5GS-06A High TIPscore

-8.67152988723384 TCGA-EB-A42Z-01A Low TIPscore

-8.99945819236197 TCGA-EB-A42Y-01A Low TIPscore

-7.81567536377316 TCGA-Z2-AA3S-06A Low TIPscore

-2.04261292232323 TCGA-ER-A3EV-06A Low TIPscore

-11.2500764267278 TCGA-EB-A5FP-01A Low TIPscore

-7.50199286153883 TCGA-FW-A3TU-06A Low TIPscore

-4.84622758445292 TCGA-FS-A4FD-06A Low TIPscore

17.2377696118 TCGA-EE-A2GE-06A High TIPscore

-13.8052686078798 TCGA-FS-A4F9-06A Low TIPscore

1.8382133602501 TCGA-FR-A3R1-01A High TIPscore

2.49872016457518 TCGA-EB-A3XF-01A High TIPscore

13.4247926949777 TCGA-WE-A8K4-01A High TIPscore

7.19143020770334 TCGA-YD-A9TA-06A High TIPscore

23.0006659313961 TCGA-EB-A5SG-06A High TIPscore

2.52919779183771 TCGA-DA-A95V-06A High TIPscore

-6.22631508427649 TCGA-FS-A1YX-06A Low TIPscore

-2.52672882328693 TCGA-EB-A3XE-01A Low TIPscore

-8.53131018370914 TCGA-D9-A4Z5-01A Low TIPscore

6.15681210427734 TCGA-D3-A2JN-06A High TIPscore

-15.5982015358811 TCGA-WE-A8ZQ-06A Low TIPscore

-7.59821338228879 TCGA-EB-A1NK-01A Low TIPscore

-5.90030979866161 TCGA-ER-A19F-06A Low TIPscore

-11.7183401573275 TCGA-EB-A3HV-01A Low TIPscore

-2.74792903650228 TCGA-BF-AAP2-01A Low TIPscore

-13.3822305582973 TCGA-EB-A6QZ-01A Low TIPscore

12.3752259661812 TCGA-D9-A3Z1-06A High TIPscore

-15.9335426040679 TCGA-FS-A1ZC-06A Low TIPscore

7.93676399546419 TCGA-Z2-AA3V-06A High TIPscore

17.6997858647413 TCGA-D3-A2JH-06A High TIPscore

-14.9463108974265 TCGA-D3-A5GT-01A Low TIPscore

21.7023291623568 TCGA-D3-A8GD-06A High TIPscore

5.00391493560173 TCGA-EE-A29M-06A High TIPscore

12.3317813605912 TCGA-EB-A4IS-01A High TIPscore

4.1726142828413 TCGA-FR-A3YN-06A High TIPscore

-3.72213916755189 TCGA-BF-A5ES-01A Low TIPscore

-5.42928443504071 TCGA-EB-A41B-01A Low TIPscore

-0.301481485918694 TCGA-EE-A2GD-06A High TIPscore

9.09055010186133 TCGA-ER-A195-06A High TIPscore

15.3174760231297 TCGA-DA-A1I5-06A High TIPscore

-14.5675556754016 TCGA-BF-A1PV-01A Low TIPscore

-12.8571212458509 TCGA-EE-A29Q-06A Low TIPscore

-11.4226055542493 TCGA-FR-A2OS-01A Low TIPscore

17.0902524582991 TCGA-ER-A2NG-06A High TIPscore

-8.76831281940527 TCGA-W3-AA1O-06A Low TIPscore

12.2356301365639 TCGA-WE-A8K1-06A High TIPscore

8.98719867845057 TCGA-EE-A2GU-06A High TIPscore

-17.2193831301711 TCGA-EE-A29L-06A Low TIPscore

13.7778595045341 TCGA-D3-A2JL-06A High TIPscore

-4.15329352315967 TCGA-EE-A2MM-06A Low TIPscore

6.28125141189207 TCGA-D9-A148-06A High TIPscore

-10.1210110580052 TCGA-LH-A9QB-06A Low TIPscore

-8.82121535685994 TCGA-EB-A5SE-01A Low TIPscore

-4.74572456561031 TCGA-WE-A8ZR-06A Low TIPscore

-5.04984443588239 TCGA-FS-A1ZJ-06A Low TIPscore

-10.7112449246919 TCGA-XV-AAZW-01A Low TIPscore

3.93672729485587 TCGA-D3-A8GE-06A High TIPscore

11.008702255769 TCGA-D3-A2JF-06A High TIPscore

-3.19113379702916 TCGA-FS-A1ZR-06A Low TIPscore

-10.3978487894006 TCGA-EE-A29B-06A Low TIPscore

6.903514250389 TCGA-FR-A7U9-06A High TIPscore

-11.6311991231795 TCGA-EB-A3Y7-01A Low TIPscore

22.2015764973481 TCGA-EE-A2M8-06A High TIPscore

8.35115959043042 TCGA-EE-A29N-06A High TIPscore

-5.30377195452284 TCGA-EE-A2MQ-06A Low TIPscore

-11.5790644668419 TCGA-FS-A1ZZ-06A Low TIPscore

-0.57953133064887 TCGA-D3-A2JD-06A High TIPscore

-7.43762441063928 TCGA-EE-A182-06A Low TIPscore

9.9619307016023 TCGA-EE-A2A6-06A High TIPscore

-12.2747208452312 TCGA-ER-A2NE-06A Low TIPscore

13.4766739417401 TCGA-GN-A9SD-06A High TIPscore

-2.09973916628422 TCGA-XV-AAZY-01A Low TIPscore

0.974035668268221 TCGA-ER-A198-06A High TIPscore

-8.38911000458017 TCGA-D9-A4Z6-06A Low TIPscore

-1.20777411773657 TCGA-ER-A2NF-06A High TIPscore

-14.0449292406482 TCGA-FS-A1ZG-06A Low TIPscore

-9.77005691165791 TCGA-FW-A3I3-06A Low TIPscore

-9.79466196790472 TCGA-EB-A4OY-01A Low TIPscore

-13.617947625726 TCGA-EB-A5KH-06A Low TIPscore

13.9221591572611 TCGA-EB-A85J-01A High TIPscore

-2.05809327147483 TCGA-D3-A1QA-07A Low TIPscore

-13.1528681575527 TCGA-EE-A29V-06A Low TIPscore

2.1151803535947 TCGA-EE-A2GT-06A High TIPscore

-13.2468958822381 TCGA-GN-A268-06A Low TIPscore

-14.2146825132094 TCGA-EE-A3AH-06A Low TIPscore

13.0705627205881 TCGA-EB-A6L9-06A High TIPscore

12.1836585120302 TCGA-EE-A2M5-06A High TIPscore

2.54680696326939 TCGA-D3-A1Q3-06A High TIPscore

-11.8043222963676 TCGA-WE-A8ZT-06A Low TIPscore

7.14447670831205 TCGA-D9-A1JW-06A High TIPscore

20.1972183676853 TCGA-EE-A2MC-06A High TIPscore

-9.39696548483501 TCGA-BF-A3DL-01A Low TIPscore

-16.0640723002102 TCGA-EE-A29A-06A Low TIPscore

13.3401763404135 TCGA-FR-A728-01A High TIPscore

-8.19120924053356 TCGA-D3-A2JG-06A Low TIPscore

24.8930934537673 TCGA-W3-AA1W-06A High TIPscore

-8.35592696694938 TCGA-EB-A6QY-01A Low TIPscore

-8.04021807329002 TCGA-BF-A3DN-01A Low TIPscore

7.68179386944235 TCGA-D3-A2JC-06A High TIPscore

6.93964463977245 TCGA-FS-A1ZM-06A High TIPscore

4.75764603910115 TCGA-QB-A6FS-06A High TIPscore

-11.033223327011 TCGA-EE-A2A5-06A Low TIPscore

-10.2479739670002 TCGA-DA-A1I8-06A Low TIPscore

-5.99167403610431 TCGA-DA-A1I7-06A Low TIPscore

18.8430745153459 TCGA-D3-A3CB-06A High TIPscore

-9.36438533486054 TCGA-D9-A4Z2-01A Low TIPscore

-4.92273974745297 TCGA-WE-AA9Y-06A Low TIPscore

2.61143553858024 TCGA-DA-A3F5-06A High TIPscore

7.3561076489878 TCGA-EE-A2GL-06A High TIPscore

11.8506231951418 TCGA-DA-A1HV-06A High TIPscore

26.584258001944 TCGA-D3-A51F-06A High TIPscore

6.20262406274562 TCGA-ER-A19Q-06A High TIPscore

9.55934504263466 TCGA-FR-A8YE-06A High TIPscore

6.46071634454385 TCGA-D3-A5GR-06A High TIPscore

8.01096490109355 TCGA-BF-AAP4-01A High TIPscore

-1.47739568058969 TCGA-W3-AA1Q-06A Low TIPscore

-14.6905066740387 TCGA-3N-A9WB-06A Low TIPscore

-14.0316803629087 TCGA-D9-A6EG-06A Low TIPscore

16.335984789005 TCGA-EE-A2MJ-06A High TIPscore

-14.5884513813155 TCGA-D3-A8GK-06A Low TIPscore

-8.04514379386808 TCGA-BF-A3DM-01A Low TIPscore

15.7130923251155 TCGA-EE-A2MR-06A High TIPscore

-5.59670411106572 TCGA-FS-A1ZQ-06A Low TIPscore

-14.1625450855406 TCGA-EB-A24C-01A Low TIPscore

3.83079400307708 TCGA-RP-A693-06A High TIPscore

22.8741250200912 TCGA-EE-A3JE-06A High TIPscore

2.83434849128411 TCGA-W3-AA1V-06B High TIPscore

-6.33251578041121 TCGA-EB-A3XC-01A Low TIPscore

31.7181219459162 TCGA-FR-A7UA-06A High TIPscore

-17.4362190580954 TCGA-FS-A1ZK-06A Low TIPscore

1.0576092526174 TCGA-D3-A51T-06A High TIPscore

7.6236708944876 TCGA-FS-A1Z0-06A High TIPscore

-10.931133892988 TCGA-EE-A20H-06A Low TIPscore

20.9053392680471 TCGA-WE-AAA4-06A High TIPscore

-1.88124215562731 TCGA-W3-A828-06A Low TIPscore

-8.59375680560106 TCGA-D3-A3C6-06A Low TIPscore

3.16437956552344 TCGA-D3-A2JP-06A High TIPscore

-8.20399162533448 TCGA-D3-A1Q4-06A Low TIPscore

-10.3368998176317 TCGA-FS-A1ZN-01A Low TIPscore

1.19650726108154 TCGA-RP-A694-06A High TIPscore

12.2911420286317 TCGA-W3-A825-06A High TIPscore

-10.3048765080335 TCGA-FS-A4F2-06A Low TIPscore

-9.72447272523866 TCGA-FS-A1YW-06A Low TIPscore

21.78630508975 TCGA-DA-A3F8-06A High TIPscore

-8.94688556031783 TCGA-DA-A95Z-06A Low TIPscore

-9.90121623806406 TCGA-FS-A1Z3-06A Low TIPscore

-6.26936515318125 TCGA-ER-A19E-06A Low TIPscore

23.5160382975392 TCGA-D3-A2J9-06A High TIPscore

15.6733541879065 TCGA-EB-A6R0-01A High TIPscore

-8.81843978679358 TCGA-EE-A3J7-06A Low TIPscore

10.9492958375102 TCGA-FS-A1ZW-06A High TIPscore

-6.11479803794089 TCGA-EB-A4P0-01A Low TIPscore

-12.3051313868193 TCGA-RP-A690-06A Low TIPscore

-14.4970978282002 TCGA-EE-A29W-06A Low TIPscore

-13.5243144024681 TCGA-FS-A1YY-06A Low TIPscore

-4.79418147131763 TCGA-DA-A3F3-06A Low TIPscore

21.3151083697198 TCGA-ER-A19P-06A High TIPscore

22.7749591700687 TCGA-DA-A1IB-06A High TIPscore

16.7591759808408 TCGA-EE-A2GH-06A High TIPscore

-17.3972153245201 TCGA-EE-A20C-06A Low TIPscore

-9.9876654429823 TCGA-ER-A19B-06A Low TIPscore

-2.90624939210279 TCGA-WE-AAA0-06A Low TIPscore

-12.4488954243899 TCGA-YD-A89C-06A Low TIPscore

1.47423786405815 TCGA-DA-A1I2-06A High TIPscore

-8.59240757179919 TCGA-EE-A29E-06A Low TIPscore

0.0717435605901089 TCGA-BF-A5EO-01A High TIPscore

16.5436317694052 TCGA-EE-A2GK-06A High TIPscore

0.859078778845587 TCGA-FS-A1ZA-06A High TIPscore

15.3442820324661 TCGA-ER-A19M-06A High TIPscore

3.1095218218197 TCGA-EB-A5UM-01A High TIPscore

-7.75548265152064 TCGA-EE-A180-06A Low TIPscore

-14.464352428792 TCGA-FR-A7U8-06A Low TIPscore

2.69076492226402 TCGA-Z2-A8RT-06A High TIPscore

20.7453395285699 TCGA-EE-A3JH-06A High TIPscore

9.74782437606374 TCGA-ER-A19G-06A High TIPscore

-8.16129742829637 TCGA-EE-A3JI-06A Low TIPscore

-18.218610831958 TCGA-BF-A1PU-01A Low TIPscore

9.30037838802976 TCGA-EE-A2A1-06A High TIPscore

-13.040016545209 TCGA-BF-AAP1-01A Low TIPscore

12.5135569866456 TCGA-D3-A3BZ-06A High TIPscore

9.23983405130012 TCGA-QB-AA9O-06A High TIPscore

-13.0885349552611 TCGA-ER-A19L-06A Low TIPscore

17.2732886008414 TCGA-D3-A1QB-06A High TIPscore

-9.41888927750862 TCGA-EE-A29C-06A Low TIPscore

-5.9363059296228 TCGA-BF-AAP8-01A Low TIPscore

16.7675840978115 TCGA-D3-A3MR-06A High TIPscore

19.2267015826697 TCGA-D3-A2JO-06A High TIPscore

7.29659451091983 TCGA-D3-A3CF-06A High TIPscore

23.6257577388996 TCGA-GF-A4EO-06A High TIPscore

-11.4446802227425 TCGA-D3-A3MV-06A Low TIPscore

-12.0652722099565 TCGA-YG-AA3P-06A Low TIPscore

16.206522404411 TCGA-GN-A26C-01A High TIPscore

8.3921129966646 TCGA-D3-A8GS-06A High TIPscore

-0.346601013331396 TCGA-FW-A3R5-06A High TIPscore

-7.97537450345543 TCGA-EB-A5SH-06A Low TIPscore

-6.18053353168624 TCGA-FS-A4F5-06A Low TIPscore

-5.49135618235925 TCGA-ER-A19T-06A Low TIPscore

-13.4661270324388 TCGA-EE-A3AD-06A Low TIPscore

-12.6451560520291 TCGA-GN-A4U3-06A Low TIPscore

-3.5827984151494 TCGA-DA-A1I0-06A Low TIPscore

-7.01337312168313 TCGA-EE-A2GB-06A Low TIPscore

-14.0568130634469 TCGA-D3-A2JE-06A Low TIPscore

-13.196049771693 TCGA-FS-A1ZD-06A Low TIPscore

-9.67657301985803 TCGA-EE-A3AG-06A Low TIPscore

-6.67757398752862 TCGA-EB-A24D-01A Low TIPscore

-3.98930995240906 TCGA-DA-A95W-06A Low TIPscore

22.7241348267658 TCGA-D3-A8GJ-06A High TIPscore

4.12730327035794 TCGA-D3-A2JA-06A High TIPscore

4.26057698990102 TCGA-EE-A2MN-06A High TIPscore

7.07894583047815 TCGA-3N-A9WD-06A High TIPscore

22.6748377640739 TCGA-EE-A2ME-06A High TIPscore

-13.603853453859 TCGA-BF-A9VF-01A Low TIPscore

-2.43065315284202 TCGA-EB-A3XB-01A Low TIPscore

-17.6306033142623 TCGA-EE-A2GR-06A Low TIPscore

-3.6487503650699 TCGA-EB-A550-01A Low TIPscore

21.1964901972047 TCGA-ER-A19W-06A High TIPscore

11.3686271351264 TCGA-GN-A4U8-06A High TIPscore

11.5375914525837 TCGA-FS-A4F8-06A High TIPscore

4.16614916435967 TCGA-ER-A19H-06A High TIPscore

-5.36094870111817 TCGA-D3-A1QA-06A Low TIPscore

14.3599375586272 TCGA-ER-A197-06A High TIPscore

-6.81743425173313 TCGA-DA-A1IC-06A Low TIPscore

-0.513177581338551 TCGA-EE-A20I-06A High TIPscore

-5.45786662364258 TCGA-D3-A8GQ-06A Low TIPscore

11.1638755244362 TCGA-EE-A181-06A High TIPscore

-10.1242815011293 TCGA-FR-A8YD-06A Low TIPscore

-10.4136686148844 TCGA-EE-A29D-06A Low TIPscore

1.97820384803681 TCGA-GF-A6C8-06A High TIPscore

-16.9046663915679 TCGA-FS-A1ZY-06A Low TIPscore

8.15171590846967 TCGA-W3-A824-06A High TIPscore

15.3411555722183 TCGA-3N-A9WC-06A High TIPscore

2.6675151829817 TCGA-W3-AA1R-06A High TIPscore

-1.30415942570837 TCGA-ER-A3ET-06A High TIPscore

-11.7887370313461 TCGA-D3-A3MO-06A Low TIPscore

-11.3014924849217 TCGA-FW-A5DX-01A Low TIPscore

-8.20946989469139 TCGA-ER-A19J-06A Low TIPscore

21.3142868485947 TCGA-FR-A44A-06A High TIPscore

3.77478729168084 TCGA-D3-A2J7-06A High TIPscore

-12.8719991589567 TCGA-EB-A5VU-01A Low TIPscore

-9.50468511070115 TCGA-D3-A1Q5-06A Low TIPscore

3.09780433486908 TCGA-WE-AAA3-06A High TIPscore

7.37666011643413 TCGA-D3-A5GO-06A High TIPscore

-6.89391051906066 TCGA-FS-A1Z7-06A Low TIPscore

-0.679999520207855 TCGA-FS-A1ZB-06A High TIPscore

-10.7916794484361 TCGA-D3-A8GI-06A Low TIPscore

6.1244015739818 TCGA-EE-A2A0-06A High TIPscore

-16.687938247117 TCGA-EB-A5SF-01A Low TIPscore

2.02668640326874 TCGA-EE-A184-06A High TIPscore

-6.9483496816373 TCGA-EE-A17Y-06A Low TIPscore

-10.2477227171134 TCGA-ER-A42K-06A Low TIPscore

2.06008859435772 TCGA-D3-A1Q6-06A High TIPscore

19.4572205623132 TCGA-GN-A265-06A High TIPscore

-10.0024011388335 TCGA-GN-A26D-06A Low TIPscore

2.48193602162005 TCGA-ER-A2NF-01A High TIPscore

-2.96046589745323 TCGA-ER-A2ND-06A Low TIPscore

-13.9803598681518 TCGA-EE-A185-06A Low TIPscore

16.426088511073 TCGA-BF-AAP0-06A High TIPscore

2.57758714048132 TCGA-EB-A5UN-06A High TIPscore

3.45767987123972 TCGA-D3-A5GN-06A High TIPscore

-11.102745337792 TCGA-D3-A8GV-06A Low TIPscore

-16.2005945364463 TCGA-BF-A5ER-01A Low TIPscore

15.1746117893974 TCGA-GF-A3OT-06A High TIPscore

13.9189882933089 TCGA-EB-A551-01A High TIPscore

-7.78872573609079 TCGA-EE-A29R-06A Low TIPscore

10.5141399647355 TCGA-EE-A2MK-06A High TIPscore

-2.39024784491565 TCGA-EE-A29G-06A Low TIPscore

3.20898402856737 TCGA-EE-A20F-06A High TIPscore

-8.6660960542785 TCGA-GN-A262-06A Low TIPscore

9.00720044395074 TCGA-EB-A85I-01A High TIPscore

11.2848694972349 TCGA-DA-A1HW-06A High TIPscore

12.6937223871227 TCGA-ER-A42L-06A High TIPscore

-19.0146731704744 TCGA-D3-A3ML-06A Low TIPscore

15.2413985820324 TCGA-DA-A3F2-06A High TIPscore

-5.23114270074913 TCGA-DA-A1I4-06A Low TIPscore

-10.8360911159113 TCGA-GN-A4U4-06A Low TIPscore

-1.16472830382301 TCGA-EE-A29S-06A High TIPscore

7.80731186879401 TCGA-WE-A8ZX-06A High TIPscore

32.9786582156636 TCGA-D3-A3C8-06A High TIPscore

10.3863626597913 TCGA-YG-AA3N-01A High TIPscore

2.44689394701577 TCGA-D3-A1Q9-06A High TIPscore

3.97720865877855 TCGA-WE-A8ZO-06A High TIPscore

15.3878148183687 TCGA-EB-A44Q-06A High TIPscore

-3.0539879363689 TCGA-GN-A8LN-01A Low TIPscore

1.17669984409572 TCGA-D9-A3Z4-01A High TIPscore

-7.74041774929454 TCGA-DA-A1HY-06A Low TIPscore

14.2839004628994 TCGA-GN-A4U5-01A High TIPscore

-9.13597938858154 TCGA-WE-A8K6-06A Low TIPscore

-12.219560533098 TCGA-DA-A95Y-06A Low TIPscore

-14.569925560333 TCGA-GF-A2C7-01A Low TIPscore

7.90144732716827 TCGA-EB-A4OZ-01A High TIPscore

-0.423486616991809 TCGA-EE-A2MS-06A High TIPscore

-6.76403969183982 TCGA-BF-A1PZ-01A Low TIPscore

-1.24920210598369 TCGA-EE-A2GS-06A High TIPscore

-6.34175173099637 TCGA-D3-A1Q8-06A Low TIPscore

2.33364143631016 TCGA-BF-A3DJ-01A High TIPscore

11.8289395920627 TCGA-ER-A19N-06A High TIPscore

2.03993283251948 TCGA-EB-A3XD-01A High TIPscore

7.00953873072423 TCGA-D3-A3C7-06A High TIPscore

13.4353850044827 TCGA-BF-AAP7-01A High TIPscore

13.963034514538 TCGA-EE-A2MU-06A High TIPscore

-13.2717155289895 TCGA-D3-A3CC-06A Low TIPscore

14.2040170968656 TCGA-ER-A1A1-06A High TIPscore

21.4958932873263 TCGA-ER-A2NH-06A High TIPscore

14.5734699313504 TCGA-D3-A51E-06A High TIPscore

-4.67555326425546 TCGA-IH-A3EA-01A Low TIPscore

-15.4425927129037 TCGA-FS-A4F0-06A Low TIPscore

9.71319711809812 TCGA-HR-A2OG-06A High TIPscore

12.7897997364072 TCGA-EE-A2MG-06A High TIPscore

22.8797453141788 TCGA-ER-A19S-06A High TIPscore

-3.76243424628524 TCGA-ER-A3PL-06A Low TIPscore

-11.9760410139036 TCGA-ER-A19C-06A Low TIPscore

0.522178568356075 TCGA-EE-A3JB-06A High TIPscore

-3.34984837730234 TCGA-EE-A2M7-06A Low TIPscore

-4.5692748156657 TCGA-FR-A69P-06A Low TIPscore

-1.27260475987804 TCGA-D3-A51K-06A High TIPscore

-4.81210598341276 TCGA-FW-A3TV-06A Low TIPscore

-5.46606779150068 TCGA-EE-A2MT-06A Low TIPscore

15.5785011544077 TCGA-D3-A2JB-06A High TIPscore

-0.36786240269474 TCGA-EE-A20B-06A High TIPscore

-3.35945116486399 TCGA-D3-A3MU-06A Low TIPscore

9.15121578185674 TCGA-FS-A1ZH-06A High TIPscore

-10.4690792206571 TCGA-EB-A82C-01A Low TIPscore

5.39433994203622 TCGA-D9-A1JX-06A High TIPscore

-0.921740035229598 TCGA-GN-A263-01A High TIPscore

9.7355504068813 TCGA-D3-A3CE-06A High TIPscore

-14.8863361546692 TCGA-EE-A2GO-06A Low TIPscore

-4.21228692202617 TCGA-XV-AB01-06A Low TIPscore

-10.4987078793232 TCGA-D9-A6EA-06A Low TIPscore

0.0219591749199511 TCGA-ER-A19K-01A High TIPscore

-11.8009249363602 TCGA-D3-A8GR-06A Low TIPscore

2.72059876143501 TCGA-EE-A3AB-06A High TIPscore

8.64003732180894 TCGA-ER-A199-06A High TIPscore

-10.1502607675925 TCGA-EB-A4IQ-01A Low TIPscore

8.30772669729367 TCGA-D3-A51N-06A High TIPscore

2.95417749557796 TCGA-DA-A95X-06A High TIPscore

24.7212919575014 TCGA-HR-A2OH-06A High TIPscore

-3.33306404971876 TCGA-EB-A3Y6-01A Low TIPscore

-0.136684116130848 TCGA-ER-A2NB-01A High TIPscore

-0.746752875576082 TCGA-EB-A299-01A High TIPscore

16.027994149582 TCGA-EE-A3JD-06A High TIPscore

20.9326100514741 TCGA-ER-A19A-06A High TIPscore

-5.66724312296612 TCGA-W3-AA21-06A Low TIPscore

1.05260973611987 TCGA-EE-A3JA-06A High TIPscore

0.716497231211406 TCGA-D3-A5GU-06A High TIPscore

-15.5934603675155 TCGA-FS-A1ZU-06A Low TIPscore

17.9435245806659 TCGA-EE-A29P-06A High TIPscore

-4.93163637521538 TCGA-ER-A196-01A Low TIPscore

-2.72070752236096 TCGA-YG-AA3O-06A Low TIPscore

22.4587149346834 TCGA-D3-A2J8-06A High TIPscore

6.13216079865464 TCGA-EE-A2MP-06A High TIPscore

-13.6418343361309 TCGA-GN-A8LL-06A Low TIPscore

-0.585789297073894 TCGA-BF-AAOU-01A High TIPscore

13.8647578348962 TCGA-BF-A1PX-01A High TIPscore

7.49688741171984 TCGA-EB-A5UL-06A High TIPscore

-11.2827036364344 TCGA-D3-A2JK-06A Low TIPscore

4.52191339874766 TCGA-EE-A3AA-06A High TIPscore

-12.4492650901669 TCGA-EE-A17X-06A Low TIPscore

-6.85035264634717 TCGA-EB-A431-01A Low TIPscore

14.0644902937374 TCGA-ER-A193-06A High TIPscore

-4.4894736120543 TCGA-EE-A2MF-06A Low TIPscore

2.3479513445458 TCGA-EE-A29T-06A High TIPscore

-11.0503841969441 TCGA-GN-A264-06A Low TIPscore

4.089372691484 TCGA-EB-A44N-01A High TIPscore

6.47952614510677 TCGA-FW-A5DY-06A High TIPscore

-9.17731197363325 TCGA-EE-A2GN-06A Low TIPscore

-18.385690718819 TCGA-ER-A3ES-06A Low TIPscore

-17.8564326363091 TCGA-DA-A960-01A Low TIPscore

-0.722166860921093 TCGA-ER-A194-01A High TIPscore

9.42690082629336 TCGA-GN-A26A-06A High TIPscore

1.81218261243467 TCGA-EB-A44R-06A High TIPscore

6.26217233735994 TCGA-XV-AAZV-01A High TIPscore

28.690361041986 TCGA-EB-A5VV-06A High TIPscore

-4.81209317492594 TCGA-OD-A75X-06A Low TIPscore

-12.4835110566038 TCGA-GF-A769-01A Low TIPscore

-3.01530988799837 TCGA-EE-A29H-06A Low TIPscore

-0.157326119231914 TCGA-EB-A51B-01A High TIPscore

-15.9057135756292 TCGA-D9-A1X3-06A Low TIPscore

3.95602512266917 TCGA-EE-A183-06A High TIPscore

9.34198761076286 TCGA-FS-A1Z4-06A High TIPscore

-16.4896542572616 TCGA-D3-A51R-06A Low TIPscore

6.45625902625605 TCGA-D9-A149-06A High TIPscore

-8.39250333308939 TCGA-EB-A44O-01A Low TIPscore

2.20701886795918 TCGA-EE-A2A2-06A High TIPscore

10.4900898149716 TCGA-GF-A6C9-06A High TIPscore

-11.9679088922517 TCGA-D3-A5GL-06A Low TIPscore

2.84158939313449 TCGA-EE-A2MI-06A High TIPscore

-8.54360331730889 TCGA-WE-A8ZY-06A Low TIPscore

1.85817191770187 TCGA-EE-A29X-06A High TIPscore

-7.72016629625287 TCGA-WE-A8JZ-06A Low TIPscore

-5.91636105579569 TCGA-GN-A4U9-06A Low TIPscore

-2.75876796871888 TCGA-FS-A4F4-06A Low TIPscore

-9.7629996640773 TCGA-D9-A4Z3-01A Low TIPscore

19.6407599020053 TCGA-D9-A6E9-06A High TIPscore

-3.38694658211421 TCGA-ER-A2NC-06A Low TIPscore

-3.27331603130392 TCGA-EB-A97M-01A Low TIPscore

-13.30164967741 TCGA-GN-A4U7-06A Low TIPscore

-3.02546967858766 TCGA-FS-A1ZP-06A Low TIPscore

5.62542816886356 TCGA-EE-A3J5-06A High TIPscore

-12.4494495589281 TCGA-D3-A8GP-06A Low TIPscore

-4.62626493689974 TCGA-XV-A9W2-01A Low TIPscore

-8.06322538433029 TCGA-EB-A553-01A Low TIPscore

-8.69945187333986 TCGA-EE-A2GM-06B Low TIPscore

18.5264284856672 TCGA-D3-A8GN-06A High TIPscore

-15.0490976954645 TCGA-D3-A1Q1-06A Low TIPscore

3.71996807294798 TCGA-BF-A5EQ-01A High TIPscore

2.1238488331312 TCGA-EE-A2ML-06A High TIPscore

-2.33757418627598 TCGA-EE-A2GP-06A Low TIPscore

-4.68231473504575 TCGA-EE-A2M6-06A Low TIPscore

-2.18076038103841 TCGA-EE-A3J3-06A Low TIPscore

-9.14261643564865 TCGA-FS-A1ZF-06A Low TIPscore

-8.5196096924179 TCGA-D9-A6EC-06A Low TIPscore

-3.5313525261345 TCGA-FR-A8YC-06A Low TIPscore

3.3601496228883 TCGA-EB-A4XL-01A High TIPscore

4.48174364268633 TCGA-EE-A3AC-06A High TIPscore

-4.2567046683387 TCGA-EE-A3J4-06A Low TIPscore

-1.88893520393126 TCGA-BF-A1Q0-01A Low TIPscore
