# Supplementary material for: Predicting immunotherapy response in melanoma using a novel tumor immunological phenotype-related gene index
Source: Front Immunol. 2024 Mar 20;15:1343425. doi: 10.3389/fimmu.2024.1343425 (PMC10987686; doi:10.3389/fimmu.2024.1343425)
Supplement: Supplementary file 11 [file DataSheet_1.zip › Data Sheet 2.DOCX]

FGR

LAP3

CASP10

CD38

ITGAL

CACNG3

CEACAM21

CD79B

MMP25

IL32

TRAF3IP3

CD4

BTK

ZBTB32

TYROBP

ALOX5

CD6

CCDC88C

WAS

CD74

BIRC3

DEF6

PLEKHO1

TYMP

SLAMF7

PRKCH

SH2D2A

TNFRSF1B

POU2F2

DAPK2

ADAM28

LCP2

TNFRSF17

TNFRSF9

FOXP3

CYBA

LY75

PRDM1

TBXAS1

PARP12

TSPAN32

CNN2

DGKA

PRKCQ

FAM107B

SPI1

TTC7A

DAPP1

FCGR2B

ACAP1

SIDT1

DHRS9

TBX21

ATP2A3

SCARF1

ARHGAP15

ICAM3

NFKB2

APBB1IP

CST7

LAMP3

P2RY10

SP140

COL4A4

PTPRC

STK17B

CYLD

SMAP2

FCN1

PILRA

LAT2

SIRPG

SLC8B1

OAS1

GMIP

LAG3

CD209

SEL1L3

NLRP1

PSME1

CEBPE

RFFL

UNC13D

SH2D3C

BLNK

IL12RB1

GADD45B

DERL3

CYTH4

MFNG

LGALS2

CRYBB1

GRAP2

NCF4

CSF2RB

UPK3A

IL2RB

GZMH

GZMB

NFKBIA

PSME2

REC8

CD40

SLA2

HCK

PPP1R16B

ELF4

PIM2

TNFSF13B

CORO1A

CCL22

COTL1

MEFV

IL21R

ATP8B4

IL7

MAP4K1

RELB

CD37

LYL1

IL4I1

LILRB1

LILRA1

RASAL3

EBI3

DENND3

CEACAM4

SIGLEC8

CD79A

NKG7

SIGLEC5

JAK3

RASA4

GIMAP2

AKNA

TNFSF8

DDX58

GATA3

CXCL12

SPOCK2

MAP3K8

P2RX1

ICAM2

SYNGR2

CCL2

CCL8

ABI3

ABCC3

MS4A6A

IL10RA

SLC15A3

CD5

C11orf21

POU2AF1

CD69

SELPLG

BIN2

PARP11

ALDH2

OAS2

PTPN6

CLEC4A

KLRB1

DSE

MAN1A1

SOD2

VNN1

VNN2

LY86

HAVCR1

ITK

IL12B

ST8SIA4

CD86

ZAP70

CYTIP

STAT1

GNLY

IL1R1

PLEK

NCF2

AMPD1

CD2

MYCL

KMO

SLAMF1

CD48

GBP3

FASLG

RPS6KA1

TNFAIP3

CCND2

ELL2

CSF3R

PTK2B

SCPEP1

FAM117A

ADCY7

BCL2L14

CD80

CCRL2

CCR2

TNFSF10

TMEM156

CXCR4

SASH3

LAX1

LY9

SRGN

HVCN1

ARHGAP9

NCKAP1L

DBH

ZNF831

ZBP1

IL9R

NAGK

GPR18

IRF1

PTGER2

PSD4

TNFSF14

S1PR4

IGFLR1

HCST

CCR7

CFP

EVI2A

FGD3

BCL11B

ADORA2A

APOL3

IGLL1

RAC2

IRF5

WDFY4

SIGLEC9

RIPK3

FCHO1

KLHDC7B

LSP1

GMFG

THEMIS2

LILRB2

CCL25

IDO1

RFTN1

GCH1

CLEC10A

ACY3

ALOX5AP

EPSTI1

EPHB2

PRAM1

GIMAP6

GIMAP4

AMPD3

ADAMDEC1

MBD2

CD180

CD101

CMPK2

IL2RA

IL15RA

DOCK2

MYBPC3

C5AR2

OSTF1

HAVCR2

OASL

TRAFD1

PRR5L

LMO2

TESPA1

AGAP2

STX11

DYSF

SP110

WNT10A

LCP1

AOAH

MYO1G

IL10

TLR4

SIT1

CD72

PIM1

SLCO2B1

IL18BP

AOX1

STAT4

PARP9

CXCL9

PARVG

CD27

RBP5

GLIPR1

GPR84

ITGB7

RHOF

RAB20

CBLN3

PSTPIP1

ITGAX

IGSF6

NLRC5

IRF8

SKAP1

ARRB2

PIK3R5

SECTM1

VAV1

MYO1F

SIGLEC10

SLC2A5

EFHD2

SYTL1

MOB3C

C1orf162

CD53

FCRL5

PTPN7

EAF2

ILDR1

TIFA

GZMA

TNFAIP8

TNIP1

PLA2G7

DOK3

FGD2

RAB19

GPR174

IL2RG

DOK2

DNAJC5B

PAOX

FERMT3

FCGR1A

RGS18

IL18

FLI1

ARL11

KCNK13

ANKRD22

HHEX

TXNDC11

CD96

GBP5

VOPP1

TTC39B

SAMSN1

PIK3AP1

ELMO1

SLA

CLIC2

BATF

CXCL13

UBE2L6

ITGAD

NECAP2

SUSD3

IL34

CD1D

NCF1

SLAMF8

RNF166

CDC42SE2

IFNAR2

C1QC

ARHGAP27

BTG2

C1R

ACE

UBASH3A

GAB3

ITGB2

CD3G

CCR5

NBEAL2

FCRL3

IKZF3

CXCL16

SCIMP

LAPTM5

VCAM1

SLAMF6

TNFAIP8L2

ARHGAP25

NUAK2

MNDA

CTLA4

ICOS

RBM47

CCR1

DTX3L

ERAP1

SAMD3

SYTL3

TAGAP

CYBB

TMEM52B

TC2N

SMCO4

PLD4

PRKCB

CLEC4E

B2M

MEI1

GNGT2

NOD2

SNX20

DPEP2

CD3D

JSRP1

NFKBID

LAIR1

LAIR2

SLC43A2

CD300A

HID1

TMC8

CCDC88B

PNOC

PTGDR

IRF2

TAP1

RHOH

IL7R

INPP5D

SIGLEC7

PARM1

CXCL11

PTAFR

RNASE6

CD52

GPR183

GPR25

CD14

MZB1

OSCAR

FPR2

FPR1

GIMAP8

TMEM37

P2RY6

GPR82

RGS19

RASGRP4

C3AR1

CD8B

CXCR6

CLEC7A

CTSW

RASGRP1

KLHL6

RAB37

CYSLTR1

GLRX

C1QB

C1QA

XCR1

CD7

PHOSPHO1

TLR1

P2RY14

TBC1D10C

LRRC25

UCP2

BASP1

KCNA3

CD163

GRB2

GPR35

CD300LB

GIMAP7

HLA-DQB1

CIITA

PCED1B

GPBAR1

CCR8

TMEM150B

FGD6

HCLS1

BHLHA15

SSTR2

PRF1

FDCSP

P2RY13

SLC9A9

TIGIT

CHST15

P2RY8

C1S

IFNL1

CSF1R

VMO1

LCK

CAMK1D

GPR132

CCR3

ASCL2

ADAP2

CLECL1

CSF1

LPAR5

APOBR

FMNL1

ANO9

IL3RA

SOCS1

SP140L

STAC3

SNAI3

CXorf38

IKZF1

EVI2B

TRIM69

C16orf54

CD300LF

BTLA

FCAR

ARHGAP30

SPATC1

CXCR3

LILRB4

TNFRSF4

TNFRSF18

LILRA5

FPR3

CARD9

SOWAHD

TTC24

PDCD1

SELL

IDO2

KIR2DL4

NUGGC

HLA-DRB1

KIF19

SEMA4A

SIRPB2

TLR7

HSH2D

HLA-DQA1

FAM163B

IL27

C5AR1

MAP3K5

SPN

GZMM

MPEG1

PDCD1LG2

PLCG2

CLEC9A

FCGR1B

SLC29A3

CARD11

HLA-DRB5

CCDC69

RCSD1

CD247

CD3E

SGMS1

CR1

FCGR3A

MAFB

HLA-DOA

HLA-DMA

HLA-DRA

AIF1

NCR3

LST1

HLA-C

LILRB3

HLA-E

HLA-F

SLFN12L

PSMB10

SAMD9

TMSB4X

DENND1C

CLEC6A

CRIP1

GIMAP1

SIPA1

IRF9

SMTNL1

IFI30

LTA

LTB

TNF

HLA-B

NFAM1

LILRA2

PSMB9

HLA-DOB

HLA-DMB

CFB

APOBEC3D

LILRA6

CEBPA

INSL3

NAIP

SIGLEC14

IGLL5

CARD17

TIFAB

IKBKE

RASSF5

CCL5

MILR1

CCL4

PIK3R6

CCL3

STAP1

GLP2R

SNAP91

RASGRP2

SCT

SLC12A3

ABCB11

TXK

TCL1A

PAPLN

CACNA1F

CD40LG

FCER2

CLEC4M

SIGLEC6

IL23A

ART4

AICDA

BACH2

TREML2

CCR6

CDX1

PASK

CR2

BCL11A

ADRA1A

FLT3

RASL11A

FAM209A

PACSIN1

SPINK2

VPREB3

ELL3

CBFA2T3

FCRL2

PIK3C2B

VRTN

TTC9

DTX1

BLK

HEMGN

CCL21

MORN3

REM2

CHST4

ARL5C

GPA33

NT5DC4

PLAC8

SLC22A3

CRB2

ADRA2A

BANK1

GRAP

ANKRD29

GPR15

MS4A1

GHRL

CD1C

TNFRSF13C

AIRE

CXCR5

TREML1

IL24

FCAMR

FCRL4

FCRL1

DNASE1L3

IL22RA2

DEFA4

SPIC

GPR182

TMIGD2

COL4A3

SERPINA9

FAM170B

PARP15

TLR10

CNTNAP2

GCSAM

GAPT

HSF5

CD19

ZC3H12D

SHISA3

CTC1

C12orf42

C9orf139

FUT7

OR52N2

SPNS3

CLEC4G

PLGLB1

CCR4

GP1BA

KLHL33

FOXI2

FOXD4L3

P2RX2

CLEC17A

C17orf99

IL17REL

CNR2

GRAPL

PAX5

FAM177B

UGT2B17

KEL

CLEC4C

ALPK2

ARRDC5

TCL1B

COLCA2

LINGO3

C12orf77

SHISA8

LILRA4

TNFRSF13B

TDGF1

PCDHAC1

OR13A1

SPIB

SSTR3

SEMA3F

MEOX1

ARHGAP44

MRC2

DCN

HGF

EHD2

ARNTL2

TLL1

KCNQ1

GYG2

EYA2

SLC9A7

PYGM

NOTCH3

NUAK1

IL4R

CAPN6

ATP8B1

SEMA5B

MECOM

PLEKHG2

PTGS1

NRP1

GGT5

CCM2L

SYNDIG1

SRPX2

MEDAG

ZNF423

NDRG4

CRISPLD2

TUBB4A

ILVBL

TGFB1

PTN

LFNG

SERPINE1

TSPAN13

OGN

GLIS3

PDLIM1

UNC5B

COL1A1

VWF

COL12A1

SMOC2

SPARC

THBS4

RASGRF2

PCDH12

PDGFRB

RBP1

DOK1

OLFML3

RGS4

ECE1

SLC19A2

CA14

FILIP1

LTBP2

PLXDC2

GLT8D2

TSHZ3

XPNPEP2

FMOD

PTGFR

PLAU

MMP19

PDE1B

TNFAIP6

G0S2

FAM124B

F13A1

COL21A1

RUNX2

EFNB2

CD93

ID1

GLIS2

STEAP4

A4GALT

DLL4

ADCY4

USHBP1

PXDN

ZBTB46

MRPS25

PTPRE

HSPA12B

LOXL2

IRAK2

GSTM5

APLNR

PDGFRA

ADAM19

ST6GALNAC4

ANGPTL2

TFAP2A

THBS1

GIPC2

CH25H

HECW2

MMRN1

LUM

TPM1

ADAMTS10

TINAGL1

DPT

VASH2

CSRNP1

SNCA

KCNMB1

SLC16A2

CHST7

FAM171A1

TENM4

P4HA3

ESAM

TAGLN

CACNA1C

EDNRA

PLA2R1

DDAH1

THY1

ROBO4

JAM2

ERG

COLEC12

PTGIR

ITGA5

PDPN

OLFML2B

SLC16A14

COL6A3

EBF1

FNDC1

SOX17

GEM

SVEP1

HTRA1

GPR176

CLMP

CYYR1

DCHS1

MFAP4

ANPEP

MAP1A

TPM4

FILIP1L

BDKRB2

COL3A1

TSPAN5

ROR2

MN1

SMAD1

SMAGP

S1PR1

COL8A2

MRGPRF

MMRN2

CD34

CHST1

CLEC14A

B3GNT5

FIBIN

FZD8

HIC1

CD163L1

FAM20C

EXOC3L1

CDH5

C3orf80

RPH3AL

NXPH3

SPNS2

TMEM119

OLFML1

SLIT3

SORCS2

FLRT2

PDE2A

KANK3

MITF

GJA4

PEAR1

ARL4C

RFX8

SULF2

FAM110D

ARHGEF15

NOTCH4

S1PR3

ECSCR

MYZAP

DOC2B
