# Supplementary material for: Predicting immunotherapy response in melanoma using a novel tumor immunological phenotype-related gene index
Source: Front Immunol. 2024 Mar 20;15:1343425. doi: 10.3389/fimmu.2024.1343425 (PMC10987686; doi:10.3389/fimmu.2024.1343425)
Supplement: Supplementary file 11 [file DataSheet_1.zip › Data Sheet 3.DOCX]

FGR

LAP3

CASP10

CD38

ITGAL

CEACAM21

MMP25

IL32

TRAF3IP3

CD4

BTK

ZBTB32

TYROBP

ALOX5

CD6

CCDC88C

WAS

CD74

BIRC3

DEF6

PLEKHO1

TYMP

SLAMF7

PRKCH

SH2D2A

TNFRSF1B

POU2F2

DAPK2

STAP1

ADAM28

LCP2

TNFRSF17

TNFRSF9

FOXP3

LY75

PRDM1

TBXAS1

PARP12

TSPAN32

CNN2

DGKA

PRKCQ

FAM107B

SLC9A7

SPI1

TTC7A

RASGRP2

SCT

DAPP1

SLC12A3

FCGR2B

ACAP1

SIDT1

ABCB11

TBX21

NOTCH3

ATP2A3

SCARF1

TXK

ARHGAP15

ICAM3

NFKB2

IL4R

APBB1IP

CST7

LAMP3

P2RY10

SP140

COL4A4

PTPRC

STK17B

CYLD

SMAP2

FCN1

PILRA

LAT2

SIRPG

OAS1

LAG3

CD209

SEL1L3

NLRP1

PSME1

RFFL

PTGS1

BLNK

IL12RB1

GADD45B

DERL3

CYTH4

MFNG

LGALS2

CRYBB1

GRAP2

NCF4

CSF2RB

UPK3A

IL2RB

GZMH

GZMB

TCL1A

PAPLN

NFKBIA

PSME2

REC8

CD40

SLA2

HCK

PPP1R16B

PIM2

CD40LG

TNFSF13B

MEDAG

CORO1A

ZNF423

CCL22

MEFV

IL21R

ATP8B4

IL7

MAP4K1

TUBB4A

RELB

CD37

IL4I1

LILRB1

LILRA1

RASAL3

ILVBL

EBI3

TGFB1

DENND3

CEACAM4

SIGLEC8

CD79A

NKG7

SIGLEC5

JAK3

RASA4

SERPINE1

TSPAN13

GIMAP2

AKNA

TNFSF8

DDX58

GATA3

CXCL12

SPOCK2

MAP3K8

P2RX1

ICAM2

CCL2

CCL8

ABI3

COL1A1

ABCC3

MS4A6A

IL10RA

SLC15A3

CD5

C11orf21

POU2AF1

CD69

SELPLG

BIN2

PARP11

OAS2

PTPN6

CLEC4A

KLRB1

DSE

MAN1A1

SOD2

VNN1

VNN2

LY86

HAVCR1

ITK

IL12B

ST8SIA4

CD86

ZAP70

CYTIP

DOK1

STAT1

GNLY

IL1R1

PLEK

NCF2

AMPD1

CD2

KMO

SLAMF1

CD48

GBP3

FASLG

CA14

CCND2

ELL2

CSF3R

PLXDC2

PTK2B

SCPEP1

FAM117A

ADCY7

BCL2L14

CD80

CCRL2

CCR2

TNFSF10

TMEM156

CXCR4

FLT3

SASH3

LAX1

LY9

SRGN

HVCN1

ARHGAP9

NCKAP1L

DBH

FAM209A

ZNF831

ZBP1

PACSIN1

RUNX2

GPR18

IRF1

PTGER2

PSD4

TNFSF14

S1PR4

IGFLR1

HCST

CCR7

CFP

EVI2A

FGD3

BCL11B

ADORA2A

APOL3

IGLL1

RAC2

IRF5

WDFY4

SIGLEC9

RIPK3

FCHO1

KLHDC7B

LSP1

GMFG

LILRB2

CCL25

IDO1

MRPS25

GCH1

PTPRE

CLEC10A

FCRL2

ACY3

ALOX5AP

EPSTI1

PRAM1

GIMAP6

GIMAP4

AMPD3

ADAMDEC1

MBD2

CD180

IRAK2

CMPK2

IL2RA

IL15RA

DOCK2

MYBPC3

C5AR2

ADAM19

HAVCR2

OASL

TRAFD1

PRR5L

LMO2

TESPA1

AGAP2

STX11

SP110

LCP1

AOAH

MYO1G

IL10

TLR4

SIT1

CD72

TFAP2A

SLCO2B1

IL18BP

CH25H

AOX1

STAT4

PARP9

CXCL9

PARVG

CD27

RBP5

GLIPR1

GPR84

ITGB7

RHOF

REM2

CBLN3

PSTPIP1

ITGAX

IGSF6

NLRC5

IRF8

SKAP1

ARRB2

PIK3R5

SECTM1

VAV1

MYO1F

SIGLEC10

SLC2A5

MOB3C

C1orf162

CD53

FCRL5

PTPN7

CSRNP1

EAF2

ILDR1

PLAC8

TIFA

GZMA

TNFAIP8

KCNMB1

PLA2G7

DOK3

FGD2

GPR174

IL2RG

DOK2

DNAJC5B

CRB2

FERMT3

FCGR1A

RGS18

IL18

FLI1

ARL11

KCNK13

ANKRD22

HHEX

TXNDC11

CD96

ANKRD29

GBP5

VOPP1

SAMSN1

PIK3AP1

ELMO1

SLA

CLIC2

CXCL13

UBE2L6

MS4A1

GHRL

NECAP2

SUSD3

CD1D

CD1C

NCF1

SLAMF8

RNF166

CDC42SE2

IFNAR2

C1QC

ARHGAP27

BTG2

C1R

ACE

UBASH3A

GAB3

ITGB2

CD3G

CCR5

FCRL3

IKZF3

TREML1

CXCL16

SCIMP

LAPTM5

VCAM1

SLAMF6

TNFAIP8L2

ARHGAP25

FCRL1

MNDA

CTLA4

ICOS

RBM47

CCR1

DTX3L

ERAP1

SAMD3

SYTL3

TAGAP

CYBB

TMEM52B

TC2N

SMCO4

SPIC

CLMP

CYYR1

PLD4

PRKCB

CLEC4E

B2M

MEI1

GNGT2

NOD2

SNX20

DPEP2

CD3D

JSRP1

NFKBID

LAIR1

LAIR2

SLC43A2

CD300A

HID1

TMC8

CCDC88B

PNOC

PTGDR

IRF2

TAP1

RHOH

IL7R

TSPAN5

INPP5D

SIGLEC7

COL4A3

CXCL11

PTAFR

RNASE6

CD52

GPR183

CD14

MZB1

OSCAR

FPR2

FPR1

GIMAP8

TMEM37

P2RY6

GPR82

RGS19

C3AR1

CD8B

CXCR6

CLEC7A

FAM170B

CTSW

RASGRP1

KLHL6

RAB37

CYSLTR1

PARP15

GLRX

C1QB

C1QA

XCR1

CD7

PHOSPHO1

TLR10

TLR1

P2RY14

TBC1D10C

LRRC25

UCP2

GAPT

HSF5

KCNA3

CD19

CD163

GRB2

ZC3H12D

GPR35

CTC1

GIMAP7

HLA-DQB1

CIITA

PCED1B

GPBAR1

CCR8

TMEM150B

HCLS1

BHLHA15

C9orf139

FUT7

SSTR2

PRF1

FDCSP

P2RY13

SLC9A9

TIGIT

CHST15

P2RY8

C1S

IFNL1

SPNS3

NXPH3

CSF1R

VMO1

LCK

GPR132

CCR3

ASCL2

CCR4

ADAP2

CLECL1

CSF1

LPAR5

APOBR

FMNL1

ANO9

KLHL33

IL3RA

SOCS1

SP140L

STAC3

SNAI3

CXorf38

IKZF1

EVI2B

TRIM69

C16orf54

CD300LF

BTLA

FCAR

ARHGAP30

SPATC1

CXCR3

LILRB4

TNFRSF4

TNFRSF18

LILRA5

FPR3

CARD9

SOWAHD

TTC24

ARL4C

PDCD1

SELL

IDO2

KIR2DL4

GRAPL

NUGGC

HLA-DRB1

KIF19

SEMA4A

SIRPB2

TLR7

HSH2D

HLA-DQA1

FAM163B

IL27

C5AR1

MAP3K5

SPN

GZMM

MPEG1

PDCD1LG2

PLCG2

CLEC9A

FCGR1B

SLC29A3

CARD11

HLA-DRB5

CCDC69

RCSD1

CD247

CD3E

SGMS1

CR1

FCGR3A

MAFB

HLA-DOA

HLA-DMA

HLA-DRA

AIF1

NCR3

LST1

HLA-C

LILRB3

HLA-E

HLA-F

SLFN12L

PSMB10

SAMD9

TMSB4X

DENND1C

ARRDC5

CLEC6A

CRIP1

GIMAP1

SIPA1

IRF9

SMTNL1

IFI30

LINGO3

LTA

LTB

TNF

HLA-B

NFAM1

LILRA4

LILRA2

PSMB9

TNFRSF13B

HLA-DOB

HLA-DMB

CFB

APOBEC3D

LILRA6

INSL3

NAIP

SIGLEC14

IGLL5

CARD17

TIFAB

OR13A1

RASSF5

SPIB

CCL5

MILR1

CCL4

PIK3R6

CCL3

SSTR3
