# Supplementary material for: Predicting immunotherapy response in melanoma using a novel tumor immunological phenotype-related gene index
Source: Front Immunol. 2024 Mar 20;15:1343425. doi: 10.3389/fimmu.2024.1343425 (PMC10987686; doi:10.3389/fimmu.2024.1343425)
Supplement: Supplementary file 11 [file DataSheet_1.zip › Data Sheet 4.DOCX]

gene log2_OR lower.95 upper.95 p-value high low significant

DNAH8 -0.85874197663702 -1.48303505974284 -0.242302456901849 0.0054749007207317 54 82 **

TENM1 -0.984046224072211 -1.76154448999618 -0.228200977173681 0.00932157501432041 29 51 **

LAMA3 -1.08015904723119 -1.952331121892 -0.241688508340097 0.00992259277691119 22 42 **

CCDC141 -1.0683203163071 -1.95987259206661 -0.212127667226008 0.0126943286950103 21 40 *

ADGRG4 -0.879163502623938 -1.60434551315316 -0.169381704828886 0.0138180479760038 35 57 *

USP29 -1.03762486787649 -1.91253548755409 -0.195583417800207 0.0139829233223061 22 41 *

COL4A5 -0.930012212198671 -1.7222105703453 -0.159687199152682 0.0164204993393534 28 48 *

SPTA1 -0.813080949249291 -1.50508043418262 -0.133206803980898 0.0177889127859506 40 62 *

BRAF -0.668307971364068 -1.23165102289795 -0.108394379012038 0.0183224387106689 99 125 *

COL4A4 -0.79067315995679 -1.47127919085169 -0.121008655847519 0.0193956750704796 42 64 *
