# Supplementary material for: Predicting immunotherapy response in melanoma using a novel tumor immunological phenotype-related gene index
Source: Front Immunol. 2024 Mar 20;15:1343425. doi: 10.3389/fimmu.2024.1343425 (PMC10987686; doi:10.3389/fimmu.2024.1343425)
Supplement: Supplementary file 11 [file DataSheet_1.zip › Data Sheet 5.DOCX]

GBP2

GBP5

CCL8

GBP1

SRGN

STAT1

CXCL10

CD53

IRF1

HLA-DMB

EVI2B

CCL4

PLEK

B2M

CXCL11

GZMA

APOL3

PTPRC

AIF1

CYBB

GIMAP4

FCGR3A

CD2

BIRC3

HLA-DRB1

CXCL13

HLA-DRA

CXCL9

CD8A

CD48

GBP4

SLAMF8

CTSS

IL2RG

IDO1

GIMAP7

HLA-DMA

GZMB

CD74

IL2RB

CCL5

HCLS1

HLA-DOA

CTSW

LAPTM5

IL4I1

HLA-DPB1

LAG3

CD3D

HLA-DQA1

PSMB9

GZMK

LCP1

PRF1

ITGB2

APOL1

SASH3

ITGAL

HLA-DPA1

NKG7

C1QA

CD8B

GZMH

C1QC

CD27

TNFSF13B

LCK

C1QB

CD7

HLA-B

RAC2

CD3E

CORO1A

HLA-DQB1

UBD

LYZ

AIM2

FGL2

PLA2G2D

SELL

FCER1G

CD52

IL32

CST7

HLA-DRB5

MZB1

SLAMF7
